# Optimal compressed sensing reconstructions of fMRI using 2D deterministic and stochastic sampling geometries

**DOI:** 10.1186/1475-925X-11-25

**Published:** 2012-05-20

**Authors:** Oliver Jeromin, Marios S Pattichis, Vince D Calhoun

**Affiliations:** 1 Department of Electrical and Computer Engineering, University of New Mexico, Albuquerque, NM, 87131, USA; 2 Vision Systems Group, Gentex Coporation, Holland, MI, 49464, USA; 3 The Mind Research Network, 1101 Yale Boulevard, Albuquerque, NM, 87131, USA

**Keywords:** Compressive sensing, MRI, fMRI, Numerical optimization

## Abstract

**Background:**

Compressive sensing can provide a promising framework for accelerating fMRI image acquisition by allowing reconstructions from a limited number of frequency-domain samples. Unfortunately, the majority of compressive sensing studies are based on stochastic sampling geometries that cannot guarantee fast acquisitions that are needed for fMRI. The purpose of this study is to provide a comprehensive optimization framework that can be used to determine the optimal 2D stochastic or deterministic sampling geometry, as well as to provide optimal reconstruction parameter values for guaranteeing image quality in the reconstructed images.

**Methods:**

We investigate the use of frequency-space (k-space) sampling based on: (i) 2D deterministic geometries of dyadic phase encoding (DPE) and spiral low pass (SLP) geometries, and (ii) 2D stochastic geometries based on random phase encoding (RPE) and random samples on a PDF (RSP). Overall, we consider over 36 frequency-sampling geometries at different sampling rates. For each geometry, we compute optimal reconstructions of single BOLD fMRI ON & OFF images, as well as BOLD fMRI activity maps based on the difference between the ON and OFF images. We also provide an optimization framework for determining the optimal parameters and sampling geometry prior to scanning.

**Results:**

For each geometry, we show that reconstruction parameter optimization converged after just a few iterations. Parameter optimization led to significant image quality improvements. For activity detection, retaining only 20.3% of the samples using SLP gave a mean PSNR value of 57.58 dB. We also validated this result with the use of the Structural Similarity Index Matrix (SSIM) image quality metric. SSIM gave an excellent mean value of 0.9747 (max = 1). This indicates that excellent reconstruction results can be achieved. Median parameter values also gave excellent reconstruction results for the ON/OFF images using the SLP sampling geometry (mean SSIM > =0.93). Here, median parameter values were obtained using mean-SSIM optimization. This approach was also validated using leave-one-out.

**Conclusions:**

We have found that compressive sensing parameter optimization can dramatically improve fMRI image reconstruction quality. Furthermore, 2D MRI scanning based on the SLP geometries consistently gave the best image reconstruction results. The implication of this result is that less complex sampling geometries will suffice over random sampling. We have also found that we can obtain stable parameter regions that can be used to achieve specific levels of image reconstruction quality when combined with specific k-space sampling geometries. Furthermore, median parameter values can be used to obtain excellent reconstruction results.

## Background

Using compressive sensing (CS), we can recover certain noise-free signals and images exactly from limited numbers of k-space samples. In theory, to reconstruct images from a limited number of samples, we require that the signal exhibits *sparsity* and *incoherence* (lack of correlation) between the sensing basis and the transform basis used for reconstruction. For one-dimensional signals with *N* samples that are composed of *T* "spikes", perfect reconstuction can be obtained from *O*(*T* ⋅ log(*N*)) frequency-domain samples [1].

**Figure 1:**
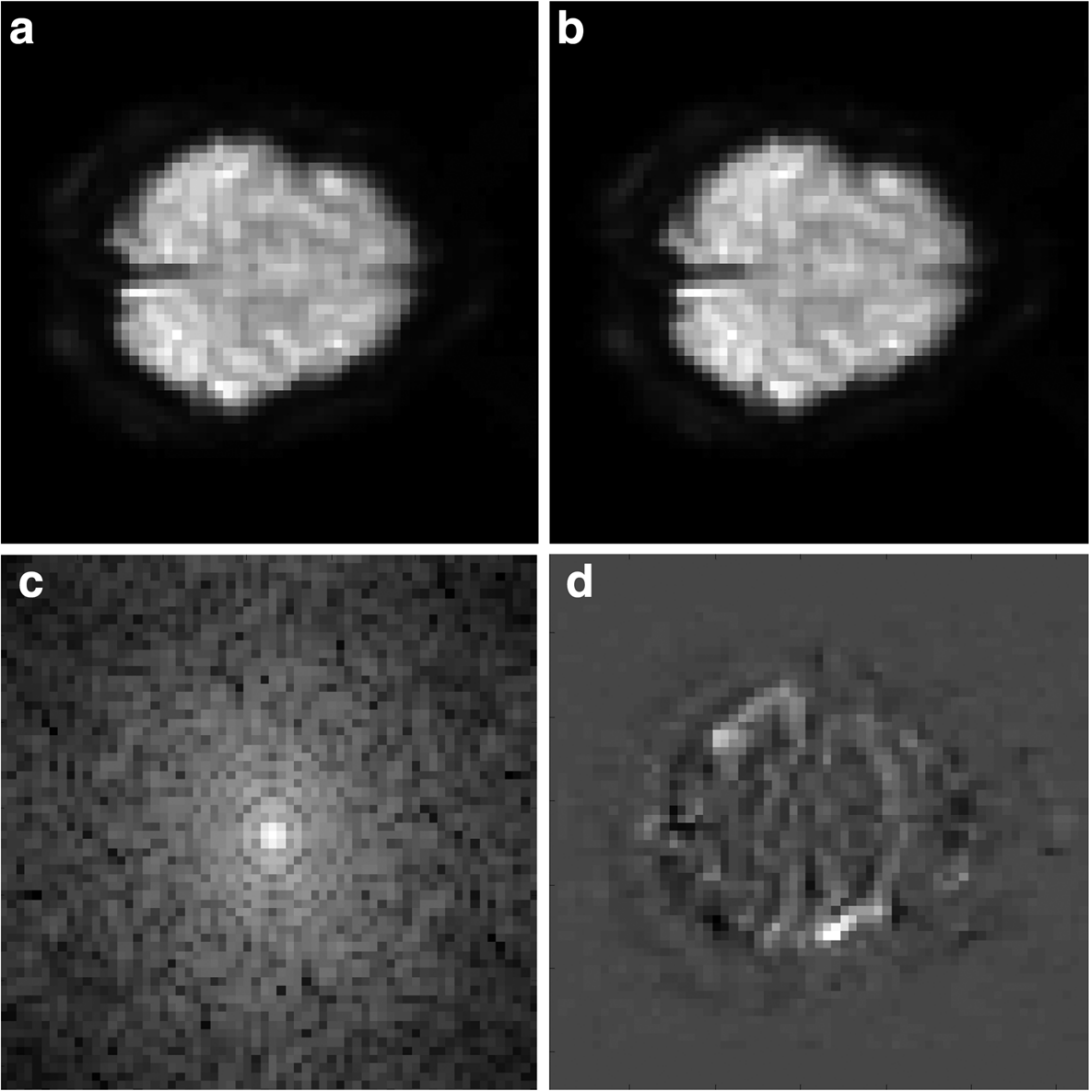
**MRI image slices with the corresponding Fourier magnitude Spectra.** Here, we approximate the Fourier magnitude spectra using a 2D FFT. (**a**) At rest (OFF) brain slice. (**b**) Active (ON) brain slice. (**c**) Log-magnitude 2D FFT of (**a**). (**d**) Difference image of both (**a**) and (**b**).

When the required conditions are met, perfect reconstruction is possible from a limited number of samples. For example, for piecewise constant signals, very impressive results have been obtained from a very limited number of Fourier samples. Unfortunately, such idealized models may not necessarily fit more complex, non-piecewise smooth images, such as standard magnetic resonance imaging (MRI) images [2]. To demonstrate the problem, we present a typical functional MRI (fMRI) slice image in Figure 1. Figure 1 depicts an fMRI data set, containing a brain slice of a patient “at rest” (OFF) and the same patient while performing a prescribed task or activity (ON). The k-space data is also depicted, revealing the energy in k-space being concentrated around the center. The difference image of the ON and OFF images, depicting the region of activity within the brain is shown in Figure 1(d).

In what follows, we demonstrate a reconstruction paradigm that explores several k-space sampling geometries. We consider both stochastic and deterministic Fourier plane sampling geometries. A limitation of our study is that it is based on retrospectively downsampled data from an original fully-sampled EPI sequence. Thus, for practical application of our findings for in-vivo experiments, the EPI sampling pattern will have to be modified to reflect the proposed downsampling patterns. Here, we note that the use of stochastic geometries that result in non-consecutive EPI phase encoding is prone to artifacts. This should not lead to problems with the SLP geometry that is found to perform best in our experiments. On the other hand, this observation can lead to reconstruction artifacts in stochastic geometries if they are implemented by moving around k-space randomly. We also note that actual MRI imaging time depends on how many phase encoding lines are acquired. Random geometries are included to comply with the conventional (sparsity and incoherence) compressive sensing requirements.

To measure the quality of the reconstructions, we use an optimization process that solves for the compressive sensing objective function parameters that maximize the peak signal to noise ratio (PSNR) or the structural similarity index matrix (SSIM) obtained by each geometry. Here, we note that the structural similarity index matrix (SSIM) [3] provides for a recent (and arguably far more reliable) image reconstruction quality metric.

Given the fact that our approach is based on numerical simulations, we avoid making claims on the actual acquisition times. We do however discuss how the most successful geometry presented here can be effectively implemented based on [4]. In [4], the authors describe a fast fMRI imaging method based on a spiral sampling geometry. As it turns out, the 2D Spiral Lowpass Sampling Geometry (SLP) that ends up performing the best among all sampling geometries in this paper represents a 2D version of the 3D *k*_*x*_*k*_*y*_*k*_*z*_ spiral sampling geometry of [4] in the 2D *k*_*x*_*k*_*y*_ plane (see Table 1 for SLP plots). This is clearly demonstrated in Figure 1 of [4]. We note that we perform 2D reconstructions on a single fMRI slice as described in the section titled “Data Set and fMRI Activity Detection,” in the “Methods” section.We also study parameter optimization for the best reconstructed image quality.We investigate the estimation of optimal parameter regions that can provide high quality reconstructions (while avoiding outliers). We investigate the relative impact of each parameter and the regularity of the optimal region. Therefore, we provide an optimization framework to help determine scanning parameters and scanning geometries that should work for the majority of the cases. In addition to parameter ranges, we also provide specific parameter values based on the median of all optimal parameter values. This approach is validated using leave-one-out for optimization of the average SSIM index.

**Table 1:** k-space sampling geometry class examples

**% Retained samples**	**62.5%**	**48.5%**	**40.6%**	**32.8%**	**28.1%**	**26.6%**	**25.0%**	**21.9%**	**20.3%**
**Geometry class**									
Dyadic Phase Encoded **(DPE)**									
Random Phase Encoded **(RPE)**									
Random Samples on a PDF **(RSP)**									
Spiral Low Pass **(SLP)**									

This table contains all of the geometric sample masks explored in this study. The columns of the table are sorted by the percentage number of samples. The rows of the table indicate a geometry class. We are interested in comparing the results of geometries across columns, as they can provide insight into the minimum number of samples that can be used that still allow us to obtain an acceptable level of SNR or mean SSIM index.

Alternatively, an optimal regularization parameter can be determined by the L-curve method [5]. Here, the “L” refers to the shape of the curve of plotting the solution norm versus the corresponding residual norm. The optimal regularization parameter is then determined by the point that gives the corner of the “L”-shaped plot. At the corner point of “L”, we have the point that minimizes the solution norm while also minimizing the residual norm. In this way, the L-curve approach avoids producing a regularization parameter that overly depends on the noise in the image [5].

We note that our parameter optimization approach is direct over the mean structural similarity index (SSIM) [3] and the PSNR. Here, we note that the PSNR is related to the residual error and it appears that the L-curve method could be applied to find robust estimates of the regularization parameters. However, the emphasis of this paper is on finding the optimal parameters with respect to the mean-SSIM [3]. We refer to [6] for a description of the problems associated with the use of the mean-squared error for digital images. On the other hand, the mean-SSIM provides for a robust metric that better correlates with human visual perception of the images that are being examined.

Our approach finds the optimal regularization parameters that minimize the mean-SSIM over a training set of images. Here, we note that each image will produce a different set of optimal parameters depending on the noise variance and how the sampling geometry captures the structure of the activity region. In the section titled “A Fourier Model for Brain Activity,” we provide a frequency-domain model for brain activity. In this section, we carefully show how brain activity favors the 2D spiral low pass geometry (SLP) in the sense that it captures most of the image energy within the effective bandwidth, while it also allows for TV-norm optimization to reconstruct the activity region.

Estimates of the optimal regularization parameters are determined by taking median values of the optimal values estimated over the training set. The effectiveness of this approach is then evaluated over an independent testing set. As we shall describe in the Results section, the approach works very well giving mean-SSIM values above 0.93 for the SLP geometry. Furthermore, note that the median is considered to be a robust, non-parametric statistic that avoids outliers [7]. The median provides for robust estimates of the regularization parameters that maximize the mean-SSIM of the reconstructed image. Here, we note the conflicting goals. Clearly, the best reconstructions will require the largest number of k-space samples. Thus, we are interested in determining the minimum acceptable quality that also yields acceptable reconstruction with the minimum number of required frequency-domain samples. To this end, we determine effective PSNR ranges and associate them with different image reconstruction qualities. For the average SSIM, we consider fixed values (SSIM > 0.75 for good results, SSIM > 0.90 for excellent results).

We limit our study to optimizing for the best MRI image reconstructions, and for the best quality activity maps based on the difference image between the “ON” and “OFF” MRI images. For detecting activity regions, we focus on the parameters that lead to highest possible quality in the difference images (activation versus no-activation images). Here, we will avoid issues associated with post-processing the difference images to better delineate between active and inactive regions of the brain. Clearly though, when the difference image is of the highest possible quality, artifact removal will simply improve upon our results. Furthermore, for very large average SSIM values (SSIM > 0.93), reconstruction quality should be considered to be excellent. For the use of post-processing methods, we refer to the body of work described in [8-15], which utilize a wide variety of signal detection, correlation analysis, and statistical tests to infer brain activity in fMRI difference images.

### Related Work

CS methods have been developed that resulted in improved spatial resolution from reduced k-space sampling. The work presented here falls into this category of k-space undersampling reconstruction methods, and is closely related to that of Lustig, Donoho, and Pauly [16]. Lustig et al. also provides a general framework for k-space sampling and reconstruction using CS theory. While the sampling geometries presented here are designed to satisfy the CS criteria, the random and pseudo-random sampling of k-space could result in slower acquisition due to scanner programming constraints.

Most of the subsequent literature on CS applications to MRI imaging explores the merging of CS theory and other fast MRI acquisition techniques, unique k-space sampling methods, and novel reconstruction algorithms. Gamper, Boesiger, and Kozerke meet the sparsity condition of CS theory by applying the Fourier transformation along the temporal dimension, assuming that some regions of the filed-of-view change at a high temporal rate while other parts remain stationary or change slowly. Their methods show the effectiveness of CS reconstruction for accelerated dynamic (continuous sampling) MRI reconstruction by comparing them to k-t BLAST reconstructions over the same data sets [17]. Their sampling scheme can be described as randomly skipping phase-encoding lines in each dynamic frame. Jung et al. developed k-t FOCUSS to provide a general framework beyond k-t SENSE and k-t BLAST for model-based dynamic MRI by applying CS theory to randomly sampled reconstruction of the prediction and residual encoding that are significantly sparse [18].

Recent studies have focused on extending the work in [16] to non-Fourier bases. Haldar, Hernando, and Liang use tailored spatially selective RF pulses to better satisfy the incoherence requirement of CS theory [19]. Liu, Zou, and Ying apply CS theory to parallel reconstruction using sensitivity encoding. Their extension of SENSE to CS is based upon a reconstruction method using Bregman iteration [20]. Trzakso, Manduca, and Borisch present a CS method that minimizes the L0 semi-norm using a re-descending M-estimator functions instead of L-1 norms typically found in CS literature [21]. The extension of the sparsity measure to multi-scale form allows for rapid reconstructions compared to the non-trivial solutions described in [17-21].

Methods that exploit the spatial and/or temporal redundancy of k-space data provide a way of describing another class of k-space undersampling reconstructions. A recent study by Lindquist et al. describes methods for obtaining a 3D echo-volumnar fMRI imaging sequence by sampling the small central portion of k-space at a high temporal rate [4]. The sampling trajectory is sampled successively across the fourth dimension in a 4D acquisition instead of successively over each temporal slice, and is constrained to a spiral pattern. Other MRI processing techniques that utilize information across slices collected at successive time intervals can be found in [22-26]. Alternatively, the authors in [27] provide a simple iterative algorithm, termed deconvolution-interpolation gridding (DING) for reconstructing MRI image from arbitrarily-sampled *k*-space. Compared to the Compressive Sensing methods discussed here, it is important to note that DING does not require regularization, and can also work with *k*-space trajectories that are not known a priori. Thus, DING is also suitable for cases where patient motion occurs during the scan.

### Contributions

The primary contributions of this paper can be summarized as follows:

• Comprehensive comparisons of 36 stochastic and deterministic frequency sampling geometries:

While the theory of compressing sensing suggests the use of stochastic geometries, we show that superior results can be achieved with deterministic frequency sampling geometries. Furthermore, we investigate the use of different sampling rates in order to determine the minimum sampling density that can still provide acceptable reconstruction results. As a consequence of this research, we provide the optimal geometry that can lead to the fastest reconstruction time among all candidate sampling geometries. This represents a contribution over existing methods that are based on a limited number of mostly stochastic sampling geometries.

• Optimal reconstruction parameter ranges for guaranteeing a minimum quality in the reconstruction images:

Current compressive sensing methods do not provide sufficient details on how to set reconstruction parameters for different applications. As a result, in current methods, there is no guidance as to which parameter values will provide reconstruction of sufficient quality. Here, we first provide a direct search optimization framework that is used to provide parameter ranges that can guarantee reconstruction of acceptable image quality. In particular, we provide parameter ranges for reconstruction BOLD fMRI ON & OFF images, as well as difference images that can be used for activity detection. Here, as evidence for the need of the proposed approach, we show that a completely different parameter range is needed for activity detection as opposed to parameters for reconstructing individual ON and OFF images. Furthermore, we found that the fast, spiral low pass sampling geometry can reach PSNR levels above 40 dB after a few iterations. In addition, the spiral low pass sampling geometry gives superior results with all reconstructions yielding mean SSIM values above 0.93.

## Methods

### Optimal TV Norm and Wavelet Transform Penalty

In what follows, we provide a summary of the proposed optimization method. As we describe below, the basic approach involves a nested optimization approach.

Let z denote the original image reconstructed using the entire k-space. Then, let *f* denote the reconstructed image. Here, *f* is a function of the reconstruction parameters *α*, *β* (further explained below) and the k-space sampling geometry, indexed by *k*. We seek to find the optimal reconstruction parameters and k-sampling geometry as given by

(1) $$\;\underset{\alpha ,\beta ,k}{\text{max}}A\left(f\left(\alpha ,\beta ,k\right),z\right)$$

where *A* denotes the accuracy of the reconstructed image. Here, we will consider two measures of accuracy: (i) the average SSIM index, and (ii) PSNR. To solve (1), we will need to solve the optimization problem of reconstructing *f* from a limited number of k-space samples.

Let *F*_*k*_ denote the k-sampling geometry operator. Let *y* denote the k-space samples that are available. In our constrained optimization approach, the reconstructed signal needs to reproduce the samples as given by

(2) $$\;{\left\|F_{k}\left(f\right)-y\right\|}_{l_{2}}\leq \varepsilon$$

where *ε* denotes a small tolerance value, and ${\left\|.\right\|}_{l_{2}}$ denotes the *l*_*2*_ norm. In (2), *F*_*k*_ takes the 2D discrete Fourier transform of the input image and forces the k-space samples in *y* to match the ones available in the *k*-th sampling geometry.

For reconstructing *f*, we follow the approach of [16,28] of seeking solutions that are sparse in the wavelet domain. This leads us to consider a wavelet reconstruction of the MRI image as given by

(3) f=Ψx

where *x* denotes the column vector of the wavelet coefficients and *Ψ* is the wavelet transform operator that contains the wavelet basis functions along its columns.

For each reconstruction geometry, we seek to find the wavelet coefficients *x* that satisfy:

(4) $$\;\underset{x}{\text{min}}\;\beta \cdot {\left\|x\right\|}_{l_{1}}+\alpha \cdot TV\left(\Psi x\right)$$

such that:

(5) $$\;{\left\|F_{k}\left(\Psi x\right)-y\right\|}_{l_{2}}\leq \varepsilon .$$

In (4), *TV*(*Ψx*) denotes the total variation of the reconstructed signal. In (4)-(5), we have a constrained optimization problem in three parameters. This is converted to an unconstrained optimization problem through the use of a Lagrange multiplier as indicated in [16,28]. In what follows, we will consider different values for *α, β* and use the software described in [16] to solve (4)-(5) for the optimal wavelet coefficients. We provide details for parameter optimization in a separate section.

### Data set and fMRI activity detection

In blood oxygenation-level dependent (BOLD) fMRI, neural activity is detected by changes in the T2^*^ relaxation time due to changes in blood oxygenation levels in response to local activation. All images were acquired on a 3 T Siemens TIM Trio system with a 12-channel radio frequency (RF) coil. The fMRI experiment used a standard Siemens gradient-echo EPI sequence modified so that it stored real and imaginary data separately. We used a field-of-view (FOV) = 240 mm, slice thickness = 3.5 mm, slice gap = 1 mm, number of slices = 32, matrix size = 64 × 64, TE = 29 ms, and TR = 2 s. The fMRI experiment used a block design with periods of 30 s OFF and 30 s ON. The subjects who participated in this study tapped the fingers of their right hand during the ON period. There were five and a half cycles, starting with OFF and ending with the OFF period.

The BOLD fMRI data was then preprocessed to account for motion artifacts and spatially normalized into the standard Montreal Neurological Institute space. This spatial normalization was then sub-sampled to 3 × 3 × 4 mm, resulting in 53 × 63 × 46 voxels. An individual slice was then selected that ensured measureable regions of activity based on the task being performed by the test subjects.

Temporal smoothing also tends to better localize detected activity across all temporal slices within a single run. Instead of utilizing the temporal information, our reconstructions are of two individual ON and OFF BOLD images. The less dense collection of temporal samples provides a worst-case scenario for detecting neural activity. We detected neural activity in fMRI by calculating the difference image of the reconstructed ON and OFF images. Individual slices were reconstructed with optimal parameters computed for different sampling geometries. At this juncture, the neural activity detection problem becomes a segmentation problem. The pre-processed sum of squares imagery was used to generate the k-space data in this study by applying the 2D Fast Fourier Transform to the multi-coil data.

As such, we will not consider optimization over a variety of different activity detection algorithms. We do note however that the activity detection algorithms that employ low-pass filtering will tend to favor zero-filling over interpolation by compressive sensing or any other method. The reason for this is that low-pass filtering attenuates high-frequency components that are estimated by the interpolation/reconstruction method. Ultimately, any segmentation for BOLD fMRI images will be governed by the accuracy of the reconstructed images themselves. Thus, for considering activity detection, we present the reconstructed difference images for qualitative comparisons. Thus, a more practical implementation of our approach would be studies involving structural MRI data, in which the benefit of a reduced acquisition time provides relief of the anxiety and discomfort patients may experience within an MRI scanner.

### A Fourier model for brain activity

In this section, we introduce an analytical model for modeling the activity (ON) image. As we shall derive in this section, the lower-frequency components can provide for an accurate model for the Fourier-Energy concentration as well as a low-TV norm image reconstruction.

**Figure 2:**
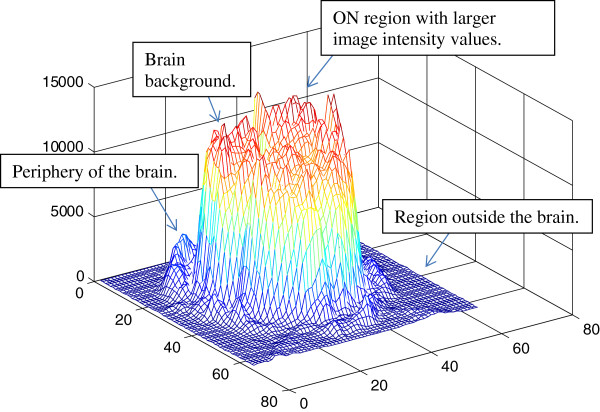
**Image model for On activity.** In this image, we show a mesh-plot of difference image #2 shown in Figure 3(b).

To develop a mathematical model for the difference image, we refer to Figure 2. There are four regions of interest: (i) the stimulated brain region (ON region) which is characterized by larger image intensity values, (ii) the brain background region that corresponds to regions that are not activated, (iii) the periphery of the brain that, and the region outside the brain. This leads to the following approximation for the spatial-domain brain image:

(6) $$\;f\left(x,y\right)\approx \{\begin{array}{ll} f_{b}(x,y), & \left(x,y\right)\;\text{is on the brain background,}\\ f_{\text{ON}}(x,y), & \left(x,y\right)\;\text{is on the stimulated region,}\\ f_{p}(x,y), & (x,y)\;\text{is on the periphery of the brain, and}\\ 0, & (x,y)\;\text{is outside the brain}. \end{array}$$

Alternatively, since the different functions do not overlap, we write:

(7) $$\;f(x,y)=f_{b}(x,y)+f_{\text{ON}}(x,y)+f_{p}(x,y)$$

Thus, our objective in reconstructing the MRI image is to effectively reconstruct the stimulated region that differentiates *f*_*ON*_ (.) from the rest. From Figure 2, we provide models for each function using a rectangular approximation for each function. The use of a rectangular approximation allows us to develop a model that is separable in the *x*-*y* coordinate system. The assumption allows us to provide analytical models and evaluate the properties of the resulting Fourier expansions for (7). Also, note that we can always express images as a sum of separable functions (e.g., via the use of the singular value decomposition or the 2D Fourier Transform).

First, we define the 1-D rectangular function rect (.) in terms of its width *W* and center C using:

(8) $$\;\text{rect}\left(x,C,W\right)=\{\begin{array}{ll} 1, & C-W/2\leq x\leq C+W/2,\\ 0, & \text{otherwise}. \end{array}$$

As mentioned earlier, for simplicity, let us assume that the brain, its periphery, and stimulated regions are all defined over rectangular regions using:

(9) $$\begin{array}{lll} \;f_{b}(x,y) & \;= & \text{rect}(x,C_{1},W_{1})\cdot \text{rect}(y,C_{2},W_{2})\cdot \left[A+a\cdot \text{cos}(\omega_{1}x)\text{cos}(\omega_{2}y)\right],\\ \;f_{\text{ON}}(x,y) & \;= & \text{rect}(x,C_{3},W_{3})\cdot \text{rect}(y,C_{4},W_{4})\cdot \left[B+b\cdot \text{cos}(\omega_{3}x)\text{cos}(\omega_{4}y)\right],\;\\ \;f_{p}(x,y) & \;= & \sum_{i=1}^{i=4}d_{i}\cdot \text{rect}\left(x,C_{3+2i},W_{3+2i}\right)\cdot \text{rect}\left(y,C_{4+2i},W_{4+2i}\right)\cdot \text{cos}\left(\omega_{3+2i}x\right)\cdot \text{cos}\left(\omega_{4+2i}y\right), \end{array}$$

where *A*≫*a*, *B*≫*b*, *A*, *B*≫*d*_*i*_ for *i* = 1, …, 4. Here, note that the background region is of image intensity given by *A* while the stimulated region is characterized by larger image intensity *B*>*A*. Over the main brain region, *f*_*b*_ is given by

$$\;A+a\cdot \text{cos}(\omega_{1}x)\text{cos}(\omega_{2}y),$$

where we have *A*≫*a*, to signify that the oscillatory components are much smaller than the average image intensity. Similarly, *B*≫*b* for the stimulated region. We can verify that both of these assumptions are valid by visual inspection of the corresponding regions in Figure 2.

We next take the Fourier-Transform of our model in (7) using:

$$\;F\left(\omega_{x},\omega_{y}\right)\;=\int_{-\infty }^{+\infty }\int_{-\infty }^{+\infty }\left[f_{b}\left(x,y\right)+f_{\text{ON}}\left(x,y\right)+f_{p}\left(x,y\right)\right]\exp\left(-j\omega_{x}x-j\omega_{y}y\right)\;dxdy.$$

To derive the final expression, note that the Fourier transform of the rectangular function is given by:

(10) $$\begin{array}{lll} \;\text{R}(\omega_{x};C,W) & \;= & F\left\{\text{rect}(x,C,W)\right\}\\ & \;= & \frac{2\text{sin}(\omega_{x}\cdot W/2)}{\omega_{x}}\cdot \exp(-jC\omega_{x})\\ & \;= & W/2\cdot \mathrm{sinc}(\omega_{x}\cdot W/(2\pi ))\cdot \exp(-jC\omega_{x}), \end{array}$$

where sinc (*ω*_*x*_) = sin(*πω*_*x*_)/(*πω*_*x*_), and **F**{.} denotes the Fourier-transform operation. Given the Fourier transform of the rectangular function in (10), the Fourier transform of its product with the cosine function simply produces two copies as given by:

(11) $$\;F\left\{\text{rect}(x,C,W)\cdot \text{cos}(\omega_{1}x)\right\}=\frac{1}{2}\left[R(\omega_{x}-\omega_{1};C,W)+\text{R}(\omega_{x}+\omega_{1};C,W)\right].$$

Using (11), we can now evaluate the Fourier Transform of our model of the brain region as given by

(12) $$\begin{array}{lll} \;F_{b}(\omega_{x},\omega_{y}) & \;= & A\cdot R(\omega_{x};C_{1},W_{1})\cdot R(\omega_{y};C_{2},W_{2})\\ & & +a\cdot R(\omega_{x}-\omega_{1};C_{1},W_{1})\cdot R(\omega_{y}-\omega_{2};C_{2},W_{2})\\ & & +a\cdot R(\omega_{x}-\omega_{1};C_{1},W_{1})\cdot R(\omega_{y}+\omega_{2};C_{2},W_{2})\\ & & +a\cdot R(\omega_{x}+\omega_{1};C_{1},W_{1})\cdot R(\omega_{y}-\omega_{2};C_{2},W_{2})\\ & & +a\cdot R(\omega_{x}+\omega_{1};C_{1},W_{1})\cdot R(\omega_{y}+\omega_{2};C_{2},W_{2}) \end{array}$$

From (12), we note that the first term *A* » *R*(*ω*_*x*_;*C*_1_,*W*_1_) » *R*(*ω*_*y*_;*C*_2_,*W*_2_) dominates the rest of the terms assuming that *ω*_1_,*ω*_2_ ≫ 0. To see this, note that the spread of the *sinc*(.)-function copies will not contribute significantly around the DC region, where the first term is concentrated. By inspection of Figure 2, it is easy to see that the assumption that *ω*_1_,*ω*_2_≫0 seems to hold. On the other hand, in the case of *ω*_1_,*ω*_2_ ≈ 0, it is easy to see that the first term still dominates since *A*≫*a*. A similar argument applies for the Fourier transform of the stimulated regions described by *f*_*ON*_ (.). In addition, note that the contributions from the periphery are limited since *A*,*B*≫*d*_*i*_ for *i* = 1, …, 4, for *i* = 1, …, 4, (refer to Figure 2 for verification). Based on these observations, we have that the Fourier Transform of the model is dominated by:

(13) $$\begin{array}{lll} \;F(\omega_{x},\omega_{y}) & \;= & F_{b}(\omega_{x},\omega_{y})+F_{\text{ON}}(\omega_{x},\omega_{y})+F_{p}(\omega_{x},\omega_{y})\approx F_{b}(\omega_{x},\omega_{y})+F_{\text{ON}}(\omega_{x},\omega_{y})\\ & & \approx A\cdot R(\omega_{x};C_{1},W_{1})\cdot R(\omega_{y};C_{2},W_{2})+B\cdot R(\omega_{x};C_{3},W_{3})\cdot R(\omega_{y};C_{4},W_{4})+\ldots , \end{array}$$

which allows us to focus on the Fourier transforms of the rectangular regions associated with the brain background region and the stimulated brain region. From (10) and (13), we note that we have the sum of two products of sinc (.) functions. Clearly, if we can identify the sinc (.) -function with the largest frequency-domain spread, we can capture most of the energy in (13). From the scaling property of the Fourier Transform, it is easy to see that the sinc (.) function with the smallest spatial-domain spread will result in the largest frequency-domain spread. Since the brain region will be larger than just the stimulated region, it is natural to assume that one of the widths associated with the stimulated region will be the smallest. Thus, without loss of generality, we assume that *W*_4_>*W*_1_, *W*_2_, *W*_3_. Furthermore, assuming that the effective spread of the sinc(.) -function extends to the three zero-crossings on either side, we have that *ω*_*spread*_ » *W*_4_/2 = 3*π* gives that *ω*_*spread*_ = 6*π*/*W*_4_. This argument can be generalized to multiple stimulated-regions. The smallest region gives the "effective" low-frequency spread as given by:

(14) $$\;\omega_{\text{effective}-\text{spread}}=6\pi /W_{\text{smallest}}$$

where W_*smallest*_ refers to the smallest stimulated region that we wish to recover in the Fourier domain. The concept of width can easily be extended to non-rectangular regions.

Now, assuming that these low-frequency components can be captured by the frequency-sampling geometry, the inverse Fourier Transform of (13) yields:

(15) $$\begin{array}{ccc} \;f_{\text{reconstructed}}(x,y) & \;\approx & A\cdot \text{rect}(x,C_{1},W_{1})\cdot \text{rect}(y,C_{2},W_{2})\\ & & +B\cdot \text{rect}(x,C_{3},W_{3})\cdot \text{rect}(y,C_{4},W_{4}) \end{array}$$

In (15), it is important to note that we have a function that has a very small TV-norm. In fact, it is an ideal case since we expect that the TV-optimized reconstruction will be well represented by the right-hand-side of (15). The observation here is that we can use the low-frequency components to capture most of the Fourier-transform energy of the ideal model as well as provide for a good initialization for optimization for low TV.

**Figure 3:**
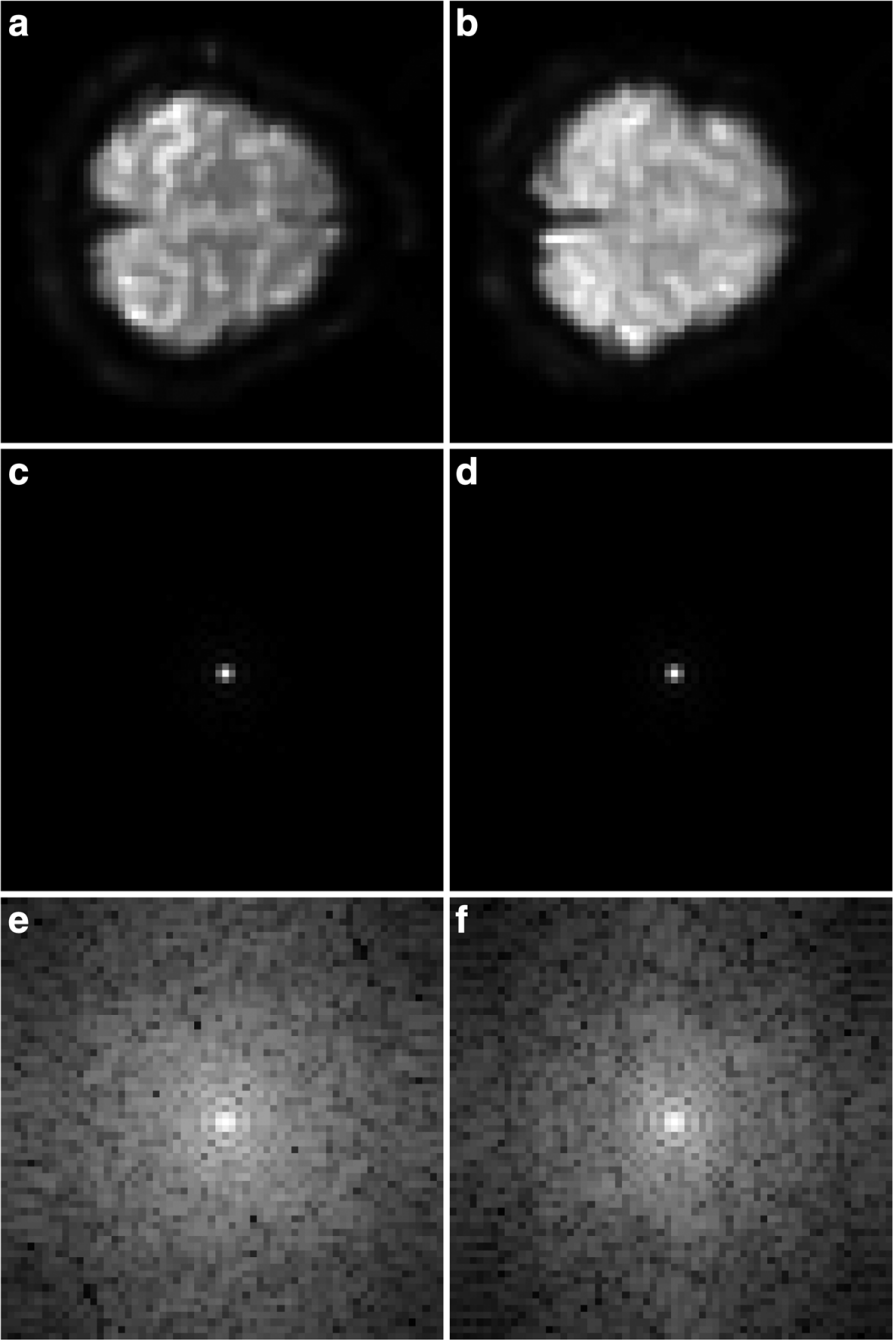
**Fourier transforms for On activity images.** (**a**) On activity image #1. (**b**) On activity image #2. (**c**) Magnitude of Fourier Transform of image #1. (**d**) Magnitude of Fourier transform of image #2. (**e**) Log-magnitude of the Fourier transform of image #1. (**f**) Log-magnitude of the Fourier transform of image #2.

The expectation that the effective Fourier magnitude frequency spread is centered around the low-frequencies is also verified experimentally in two activity images as shown in Figure 3. From Figures 3(c) and 3(d), it is quite clear that most of the energy is concentrated around the lower-frequency portion of the spectrum. The log-magnitude plots of 4(e) and 4(f) show the attenuation of the spectrum at higher frequency magnitudes. Our discussion in this section clearly shows that the low-frequency components of Figures 3(c) and 3(d) also capture the elevated image intensity levels associated with activated brain regions.

### The k-Space downsampling geometries

We consider both deterministic and stochastic sampling geometries. For deterministic geometries, we consider the spiral low-pass and dyadic downsampling along the phase encoding direction. Stochastic methods are based on random sampling.

First, we consider geometries that restrict down sampling in the phase-encoded dimension only. Here, we wish to compare k-space sampling geometries in just the phase-encoded dimension to k-space undersampling in both dimensions around the center.

As noted in the literature review, the central region of k-space is essential in obtaining reconstruction performance that is acceptable. Almost all non-CS reconstruction of k-space undersampling included the center of k-space in the data that was sampled. A dyadic sampling geometry class was developed that considers sparse sampling along the phase-encoded dimension of k-space. All geometries of this class are shown in the first row of Table 1. This geometry includes samples from a collection of contiguous frequency encoded vectors centered over the center of k-space. The width of the central region can be varied to generate a number of unique masks that are members of this mask class. Nine unique geometries are generated based on a central region that sample 1/2, 1/3, 1/4, 1/6, 1/8, 1/10, 1/12, 1/16, and 1/32 of the phase encoded samples. Beyond the bounds of the central region of the geometry, additional frequency encoded vectors are sampled every 2^nd^, 4^th^, 8^th^, etc. phase encoded sample until the entire support of the fully sampled k-space has been included. The dyadic characteristic of the gap size between subsequent high-frequency samples was intentionally designed to sample more densely near the center of k-space, and in hopes that such sampling in k-space might coincide with the wavelet transform our reconstruction method utilized. This class will be referred to as the dyadic phase encoded (DPE) geometry class in the remainder of this paper.

We also consider two additional geometry classes which attempt to increase the incoherence between the sensing and transform bases by introducing an element of randomness in how the samples were selected. The first class, which is referred to as the random phase encoded (RPE) geometry class, samples frequency-encoded vectors along random phase encoded samples. This sampling method was selected as a comparison to the dynamic CS MRI in [20]. In two images, single fMRI study, such sampling would not expect to provide acceptable reconstruction due to the possibility of excluding a portion of the central region of k-space. Each of the random phase encoded geometries contains the same number of samples as one of the dyadic phase encoded geometries. Due to the one-to-one geometry correspondence across classes, there are nine total geometries in the RPE class. All of the random phase encoded geometries are shown in the second row of Table 1.

The second geometry class that incorporates randomness into the sampling scheme can be described as random sampling along a 1-D probability distribution function across the phase encoded samples at each frequency-encoded sample. This geometry class will be referred to the random sampling PDF (RSP) class.

The motivation of this geometry is from [16], but we alter the distribution to be replicated at each frequency-encoded sample instead being defined over both k-space dimensions. We use a fifth-order polynomial of the form

(16) $$\;f\left(u,v\right)\;\propto {\left(\;1-\sqrt{u^{2}-v^{2}}\right)}^{5},\;0<u_{1},u_{2}\leq U-1,\;0<v_{1},v_{2}\leq V-1$$

where *U* and *V* are the number of k-space samples in the frequency and phase encoded dimension. This function mathematically defines the probability density function on which the random samples are being selected. Our motivation for this geometry was to attempt a compromise between the inclusion of the center of k-space and also imparting incoherence into the CS problem through a pseudo-random sampling geometry. Representative geometries of the RSP class are depicted in the third row in Table 1.

Lastly, we include a geometry class that restricts k-space sampling in both the phase encoded and frequency-encoded dimensions. Here, we generate the nine geometries by a sampling along Cartesian spiral emanating from the center of k-space. Such sampling geometries are more typical of the classical k-space undersampling reconstruction techniques found in literature. This class is referred to as the spiral low pass (SLP) geometry class (see Table 1).

### Optimization of CS penalty parameters

Parameter optimization is performed using two separate approaches. In the first approach, we want to estimate the optimal parameter ranges for *α, β* that can provide a certain level of image quality. This first approach is based on maximizing the PSNR in the reconstruction images. Our second approach is more direct. It is based on maximizing the average SSIM index. In our second approach, we want to recommend specific parameter values for each sampling geometry. The second approach is validated using leave-one-out.

Both approaches assume that we can find optimal parameters when ground truth is available. When ground truth is not available, our expectation is that our training sets will reflect the new datasets that will be collected. To initialize optimization, we first zero pad the images from the original size of 53 × 63 to be of size 64 × 64. We then use the 2D FFT to generate k-space data for our experiments. A baseline reconstruction is obtained by applying the inverse-Fourier transform to the k-space undersampling matrix. This initial reconstruction will exhibit the aliasing artifacts that the work presented in the introduction seeks to suppress. We then use the Nelder-Mead search algorithm to estimate optimal reconstruction parameters of (1) [29]. Here, we note that the Nelder-Mead algorithm does not require that we evaluate the derivatives. It belongs to the class of direct search methods [30]. This approach is used to generate a collection of optimal parameter values *α, β* associated with each image.

Then, in our first approach, we estimate the optimal parameter ranges for *α, β* that can provide a certain level of image quality. To understand how this works, note that minimum image quality requirement can be expressed as a minimum requirement for the PSNR level. We then determine the reconstructed images that exceed the minimum level. The optimal parameter region can then be estimated from the range of values of *α, β* that correspond to these images. To avoid outliers, the optimal regions need only be met by 75% of the total number of the considered images. We also consider the complexity of each optimal parameter region based on the inner and outer bounding boxes. The inner bounding box is the largest rectangle that can be totally contained inside the optimal parameter region. On the other hand, the outer bounding box represents the smallest rectangle that contains the optimal parameter region. A detailed example of our first approach is also provided in the results section.

Our second approach is more direct. Our goal is to provide specific recommendations for *α, β* that can be expected to exceed a minimum level of image quality. For this direct approach, we use the average SSIM index value to determine the required level of image quality. We consider an image with an average SSIM index value above 0.9 to represent excellent reconstruction quality. In this second approach, the image reconstruction quality is estimated using a leave-one-out method. In leave-one-out, we construct training sets consisting of all but one of the ON/OFF image pairs. We then report the reconstruction image quality on the remaining ON/OFF image pair that serves as our testing set. The method is then applied over all image pairs. Here, we use the median parameter values estimated over each training set for reconstruction of the testing set. In this approach, we report our results for each image serving as a member of the testing set. Furthermore, for simplicity, we provide the median parameter values over the entire dataset. Also, for completeness, we also provide optimal parameter values for each image pair. This allows the readers to see if the discrepancy between the median and the optimal parameter values will affect the reconstructed image quality.

## Results

We provide optimization results independently for the ON, the OFF images, as well as for the difference image. Here, we consider optimization of the difference image for activity detection.

**Figure 4:**
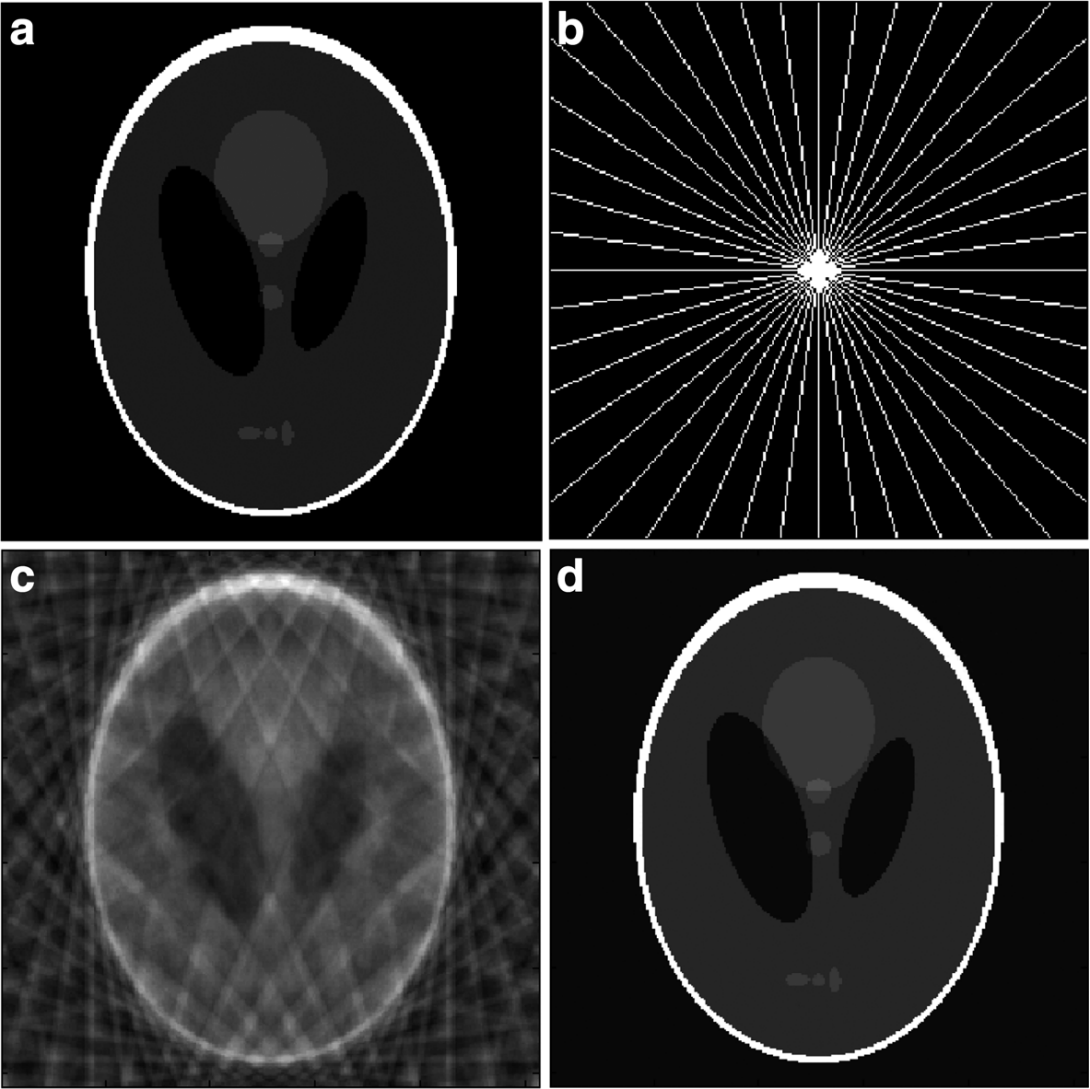
**Example of a simple CS reconstruction problem.** (**a**) The Shepp-Logan phantom test image. (**b**) The sampling geometry from which Fourier coefficients are sampled along 22 approximately radial lines. (**c**) The zero-filling result, where non-sampled coefficients are set to zero and an inverse Fourier transformation is applied. (**d**) Reconstruction obtained using CS methods. The reconstruction is an exact replica of the image in (**a**). We also tested all sampling geometries of Table 1 on this image. For sampling geometries using 20.3% of the total samples, reconstructions gave values well-above 40 dB (50-60 dB) which are excellent (e.g., see Figure 5). It is interesting to note that stochastic geometries perform significantly better than deterministic geometries at higher sampling rates. For example, random sampling on PDF gave over 90 dB reconstructions while the SLP geometry gave reconstructions of less than 60 dB. Again, all of these reconstructions are excellent. The lack of perfect-reconstruction for our geometries comes from the use of Wavelet transform in our method. As it is well-known, and also demonstrated here, the standard TV-norm is the best fit for this example.

In Figure 4, we use the Shepp-Logan phantom for testing with the classical TV optimization method of [1], as well as for the proposed Wavelet optimization framework based on [16]. As expected, we can see from Figure 4(d) that the standard TV method performs very well here. We get perfect reconstruction from just 22 radial lines shown in Figure 4(b). The stochastic sampling geometries also perform better than the deterministic geometries on this example. For example, at the highest sampling density, random sampling on PDF gives reconstructions well over 90 dB that are 30 dB above any reconstruction achieved by the SLP geometries. However, please note that this example is unrealistic in the sense that it does not capture the complexity of the activity region in fMRI.

**Figure 5:**
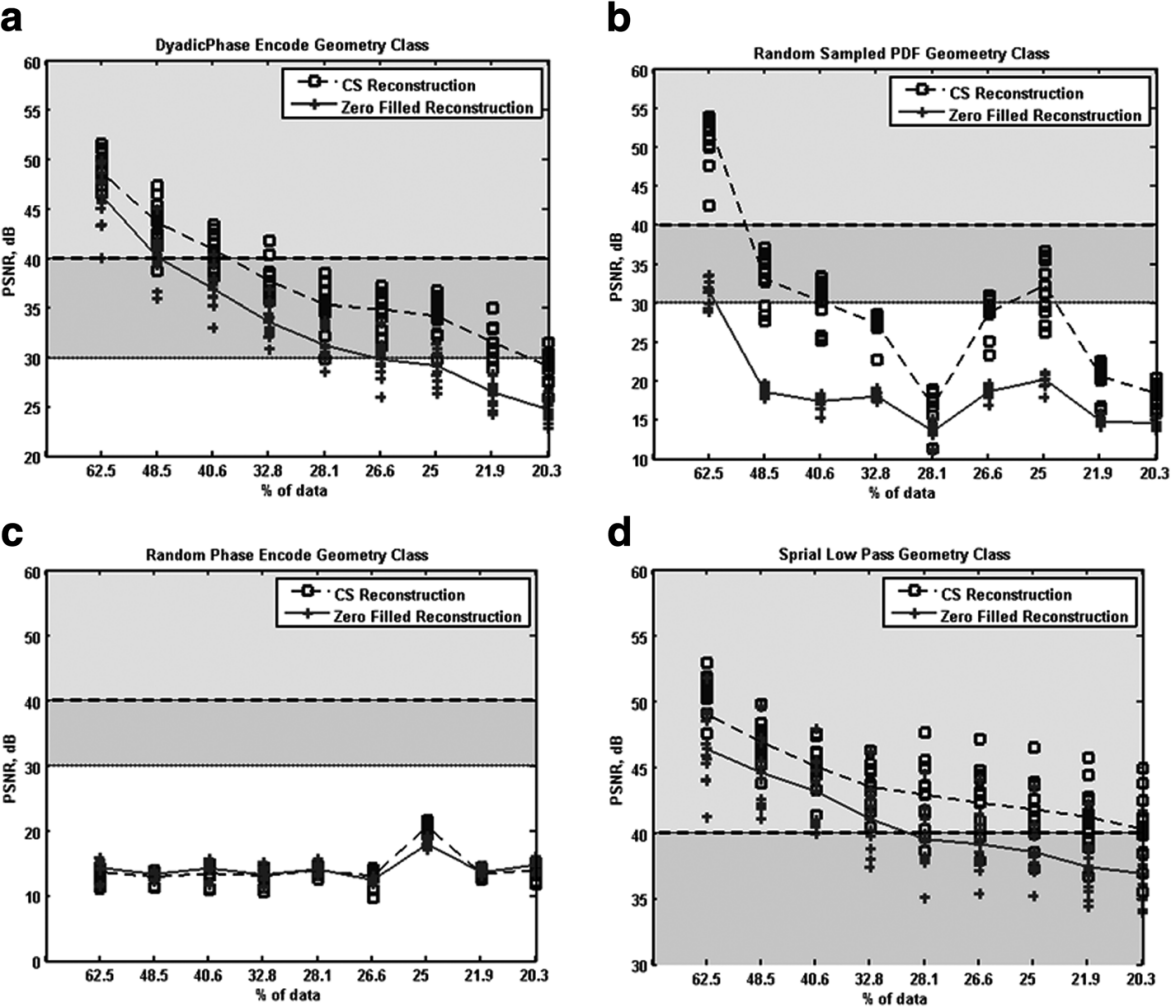
**Reconstruction image quality assessment.** The top row contains reconstructions from various SLP sampling geometries, not necessarily using the optimal reconstruction parameters. The bottom row contains reconstruction from the RSP sampling geometry class. (**a**) and (**e**) are the original ON images. (**b**) and (**f**) are marginal reconstructions of 28.35 and 24.22 dB, respectively. (**c**) and (**g**) are acceptable reconstruction of 34.97 and 39.64 dB. (**d**) and (**h**) are excellent reconstructions of 48.35 and 56.69 dB, respectively.

**Figure 6:**
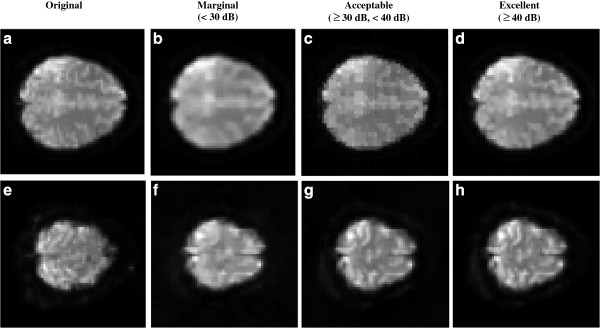
**Optimal PSNR values for CS reconstructions for each sampling geometry for both ON and OFF images.** The optimal reconstructions are represented by the ‘o’ symbol. For comparison, the ‘+’ symbol represents the PSNR achieved by simply zero-filling the missing k-space samples. The plots are also partitioned in to PSNR values that demonstrated excellent reconstruction (PSNR > 40 dB), adequate reconstruction (PSNR < 40 dB, but > 30 dB) and inadequate reconstruction (PSNR < 30 dB). (**a**) The results of sampling k-space with the DPE geometry class. (**b**) The results of the RPE geometry class. (**c**) The results of the RSP geometry class. (**d**) The results of the SLP geometry class.

For all sampling geometries, we provide optimal PSNR results for ON and OFF images in Figure 6. The PSNR values obtained by simply zero-filling the missing k-pace samples are displayed for comparison. A brief inspection reveals the PSNR values for the DPE and SLP geometry classes are higher than the RPE and RSP geometry classes. The dyadic phase encoded geometry class exhibited the most consistency between PSNR values across images for the same geometry, but had a lower average PSNR value across images than the spiral low pass geometry class. The random classes exhibited a greater improvement over zero-filling, but did not achieve reconstructed images as accurately as the deterministic classes (for the same number of samples). In terms of PSNR, the spiral low pass sampling geometry gave the best results. Furthermore, for PSNR > 40 dB, most reconstructions were found to be of excellent quality.

To determine the minimum acceptable reconstruction quality, we consider PSNR values from 20 dB to 45 dB (see Figure 5). For a given sampling geometry and a required image quality, all parameter regions were intersected to determine the maximum number of images that achieve the desired reconstruction quality for a single range of parameters. Thus, for each quality level, we can have a maximum of 24 images, indicating that the same optimal region provided this minimum quality for all of them. To avoid outliers, we consider an optimization region to be successful in meeting the image quality criterion if 75% or more of the images maintain a level above what is required. We also provide bounding box results in Table 2.

**Table 2:** Optimal parameter regions for achieving different image quality bounds

**Geometry class**	**Threshold**	**Inner bounding box**			
		**Min α**	**Max α**	**Min β**	**Max β**
*DPE 62.5%*	45 dB	8.0808e-05	2.8687e-04	2.5758e-04	3.9697e-04
*DPE 48.5%*	40 dB	1.4949e-04	2.5253e-04	2.3434e-04	3.9697e-04
*DPE 32.8%*	35 dB	2.1111e-04	3.0404e-04	3.8990e-04	4.9293e-04
*DPE 21.9%*	30 dB	4.2424e-04	6.3030e-04	2.8081e-04	3.2727e-04
*SLP 62.5%*	45 dB	2.5253e-04	4.2424e-04	1.4141e-04	1.8788e-04
*SLP 40.6%*	40 dB	1.4949e-04	2.8687e-04	7.1717e-05	2.1111e-04
*SLP 20.3%*	35 dB	8.0808e-05	1.4949e-04	4.8485e-05	9.4949e-05
*SLP 20.3%*	30 dB	1.2121e-05	1.4949e-04	2.5253e-05	9.4949e-05
**Geometry class**	**Threshold**	**Outer bounding box**			
		**Min α**	**Max α**	**Min β**	**Max β**
*DPE 62.5%*	45 dB	-2.2222e-05	3.8990e-04	1.8788e-04	4.6667e-04
*DPE 48.5%*	40 dB	4.6465e-05	3.2121e-04	1.6465e-04	5.1313e-04
*DPE 32.8%*	35 dB	1.1818e-04	3.7374e-04	1.4949e-04	5.9596e-04
*DPE 21.9%*	30 dB	1.4141e-04	3.9697e-04	2.5253e-04	7.3333e-04
*SLP 62.5%*	45 dB	4.6465e-05	4.5859e-04	7.1717e-05	3.0404e-04
*SLP 40.6%*	40 dB	2.8687e-04	4.2424e-04	2.0202e-06	3.0404e-04
*SLP 20.3%*	35 dB	-2.2222e-05	2.5253e-04	-2.1212e-05	1.8788e-04
*SLP 20.3%*	30 dB	-5.6566e-05	2.5253e-04	-2.1212e-05	2.5758e-04

This table provides bounding box parameters that are needed for achieving different performance levels.

Our experiments reveal that the DPE and SLP geometry class outperform the RPE and RSP classes. It was expected that the RPE class exhibit poor reconstruction, since the geometries do not contain sufficient samples from the center of k-space. And only a single geometry, the RSP geometry including 62.5% of samples resulted in a suggested operating parameter space for achieving “acceptable” reconstruction.

On the other hand, the DPE class achieved excellent reconstruction from two geometries (62.5% and 48.5%) and the SLP class achieved excellent reconstruction from the three geometries that retained the most number of k-space samples (60.5%, 48.5%, and 40.6%). When lower-quality reconstructions are acceptable, we can use sampling geometries that use fewer k-space samples. Twelve additional DPE geometries were found to satisfy the parameter space threshold and intersection procedure. All eighteen (nine each from the ON and OFF imagery) SLP geometries that were included in this experiment satisfied our requirement for acceptable reconstruction.

**Figure 7:**
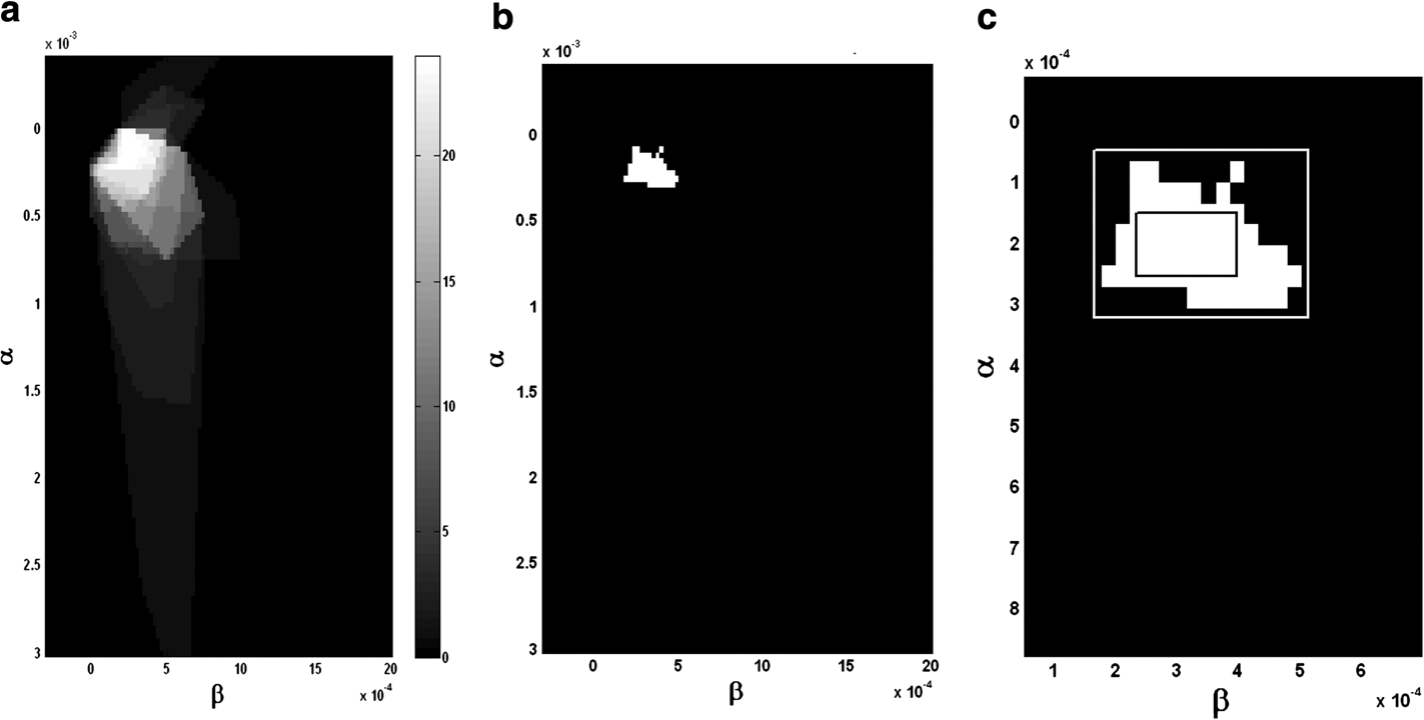
**Parameter Optimization over an entire fMRI image data set for the 48.5% DPE geometry for image quality level at 40 dB.** (**a**) Parameter region plot indicating the number of images that were reconstructed with >40 dB image quality level (max = 24). (**b**) Parameter region where 75% of the images exhibited image quality >40 dB (**c**) Inside and outside bounding boxes meant to characterize the complexity of the optimization region.

The combined parameter plots over all images indicate the number of images that meet the quality criteria. We present an example in Figure 7 for the 48.5% DPE geometry. Figure 7(c) depicts the inner and out bounding regions, where 75% of the images meet the quality requirements. The complexity of this optimal region is reflected in an area ratio of 5.71, indicating that the area of the outer box is 5.71 times larger than the inner bounding box.

For activity detection, we also compared the difference images of the original ON and OFF samples, the CS reconstructed ON and OFF samples, and also the reconstruction values of zero-filling the missing k-space samples. Based on our investigations, we have found that it was possible to obtain excellent reconstruction results with very fast k-space sampling geometries. In what follows, we present our best results, obtained with the SLP geometry using only 20.3% of k-space.

**Figure 8:**
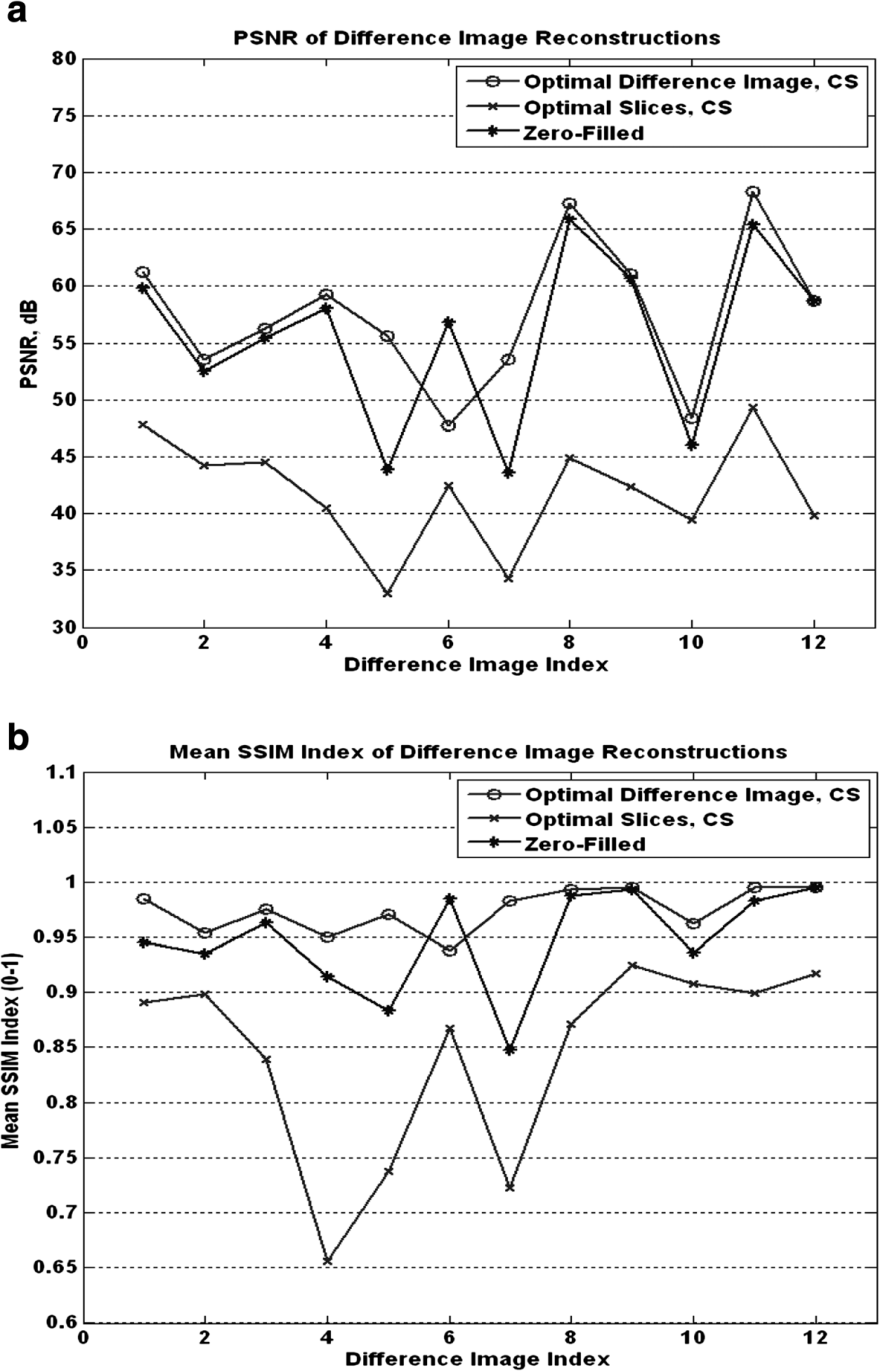
**Optimal results for fMRI activity detection for the SLP geometry using 20.3% of the samples.** (**a**) The plots of each difference image set PSNR values. (**b**) The plots of the mean SSIM index values for each difference image set. The results of the individual slice optimal CS reconstructions are shown, along with the results of the optimal difference image CS reconstructions and the zero-filled reconstruction results.

Here, it is interesting to note that activity detection required optimization of the reconstructed difference image. Separate optimization of the ON/OFF images did not translate into any improvements over simple zero filling (see Figure 8). On the other hand, optimization of the difference images led to significant increases in PSNR and SSIM over zero-filling for eleven of the twelve samples. The mean values for the optimal difference image parameters were 57.58 dB and 0.9747 for the PSNR and mean SSIM measurements, respectively. The plots in Figure 6 show the PSNR and mean SSIM values for each difference image for the optimal CS reconstruction for each slice difference image, the optimal difference image CS reconstruction, and the zero-filled difference image. For activity detection, after removing outlier points, 75% of the optimal parameter region was bounded by:

$$\begin{array}{lll} \;\alpha_{\text{min}} & \;= & 2.407\;e-7,\;\alpha_{\text{max}}=2.881\;e-5,\;\beta_{\text{min}}=9.638\;e-8,\\ \;\beta_{\text{max}} & \;= & 1.084\;e-4. \end{array}$$

We also present results from parameter optimization based on maximizing the mean SSIM index value. For each sampling geometry and image pair, we present the best mean SSIM index in Tables 3, 4, 5, 6. The corresponding *α, β* values are shown in Tables 7, 8, 9, 10, 11, 12, 13, 14. Overall, the SLP geometry gave the best results. The mean SSIM results achieved by the SLP geometry are given in Table 3. The optimal *α*-values are given in Table 8 and the optimal *β*-values are given in Table 12.

**Table 3:** Dyadic phase geometry: Mean SSIM results (OFF & ON) over each image pair and median over all pairs

**Sample rate**	**Image1**	**Image2**	**Image3**	**Image4**	**Image5**	**Image6**	**Image7**	**Image8**	**Image9**	**Image10**	**Image11**	**Image12**	**Median**
62.50	0.9938	0.9935	0.9890	0.9932	0.9937	0.9928	0.9880	0.9933	0.9922	0.9921	0.9949	0.9931	**0.99315**
48.50	0.9839	0.9806	0.9721	0.9803	0.9842	0.9781	0.9748	0.9749	0.9773	0.9784	0.9844	0.9817	**0.97935**
40.60	0.9661	0.9696	0.9512	0.9657	0.9633	0.9558	0.9575	0.9608	0.9596	0.9613	0.9668	0.9658	**0.9623**
32.80	0.9347	0.9428	0.9207	0.9358	0.9452	0.9027	0.9223	0.9371	0.9331	0.9223	0.9518	0.9422	**0.93525**
28.10	0.9032	0.9277	0.8863	0.9020	0.9173	0.8918	0.9163	0.9068	0.9024	0.8963	0.9268	0.9206	**0.905**
26.60	0.8758	0.9097	0.8612	0.8865	0.8928	0.8537	0.8440	0.8918	0.8922	0.8678	0.9068	0.8987	**0.88915**
25.00	0.8798	0.8957	0.8379	0.8891	0.8815	0.8705	0.8799	0.8935	0.8764	0.8666	0.8990	0.8972	**0.8807**
21.90	0.8452	0.8624	0.8087	0.8569	0.8394	0.8409	0.8507	0.8559	0.8483	0.8380	0.8649	0.8653	**0.8495**
20.30	0.7779	0.7816	0.7852	0.7822	0.7928	0.6842	0.6782	0.8073	0.8019	0.7844	0.8179	0.8110	**0.7848**

Average SSIM values are computed using the leave-one out method. The median parameter values of the 12 training sets are set to the median values of the 11 images in the training set and then tested on the remaining image pair. The table shows the results from the testing phase.

**Table 4:** Spiral Low Pass geometry: Mean SSIM results (OFF & ON) over each image pair and median over all pairs

**Sample rate**	**Image1**	**Image2**	**Image3**	**Image4**	**Image5**	**Image6**	**Image7**	**Image8**	**Image9**	**Image 10**	**Image 11**	**Image 12**	**Median**
62.50	0.9923	0.9938	0.9916	0.9936	0.9942	0.9929	0.9897	0.9934	0.9931	0.9941	0.9952	0.9945	**0.9935**
48.50	0.9888	0.9877	0.9878	0.9891	0.9889	0.9870	0.9801	0.9891	0.9869	0.9884	0.9908	0.9850	**0.9881**
40.60	0.9856	0.9829	0.9824	0.9848	0.9860	0.9824	0.9726	0.9860	0.9820	0.9841	0.9867	0.9791	**0.9835**
32.80	0.9789	0.9762	0.9727	0.9784	0.9811	0.9741	0.9679	0.9815	0.9761	0.9804	0.9828	0.9698	**0.9773**
28.10	0.9697	0.9659	0.9624	0.9703	0.9773	0.9649	0.9574	0.9728	0.9671	0.9731	0.9767	0.9682	**0.96895**
26.60	0.9660	0.9616	0.9541	0.9674	0.9748	0.9632	0.9552	0.9681	0.9637	0.9699	0.9718	0.9652	**0.9656**
25.00	0.9639	0.9567	0.9528	0.9643	0.9716	0.9600	0.9497	0.9657	0.9585	0.9687	0.9713	0.9640	**0.96395**
21.90	0.9512	0.9478	0.9342	0.9543	0.9630	0.9468	0.9427	0.9523	0.9456	0.9556	0.9606	0.9531	**0.95175**
20.30	0.9424	0.9356	0.9244	0.9442	0.9586	0.9285	0.9300	0.9385	0.9316	0.9467	0.9545	0.9503	**0.94045**

The results are based on the leave one out method. See Table 3 for details.

**Table 5:** Random sampling on PDF geometry: Mean SSIM results (OFF & ON) over each image pair and median over all pairs

**Sample rate**	**Image1**	**Image2**	**Image3**	**Image4**	**Image5**	**Image6**	**Image7**	**Image8**	**Image9**	**Image 10**	**Image11**	**Image 12**	**Median**
62.50	0.9917	0.3613	0.9946	0.9915	0.9965	0.4869	0.8859	0.4286	0.8429	0.9873	0.8771	0.9968	**0.9366**
48.50	0.8839	0.7967	0.3797	0.9873	0.8862	0.7779	0.9661	0.8454	0.8591	0.4017	0.8990	0.9863	**0.8715**
40.60	0.9538	0.5490	0.9520	0.9526	0.9626	0.7717	0.7206	0.9405	0.9593	0.9579	0.9668	0.9618	**0.9532**
32.80	0.9302	0.8089	0.4618	0.8772	0.8536	0.3084	0.4843	0.4917	0.9077	0.3930	0.6974	0.9397	**0.75315**
28.10	0.7025	0.8010	0.4048	0.4846	0.9073	0.6879	0.7178	0.7995	0.4558	0.6144	0.8163	0.5519	**0.6952**
26.60	0.7432	0.4272	0.4323	0.5289	0.7348	0.7207	0.3768	0.8983	0.4436	0.5258	0.8413	0.4048	**0.52735**
25.00	0.5855	0.9114	0.6392	0.5203	0.4872	0.5883	0.2242	0.8795	0.3602	0.5417	0.9151	0.5172	**0.5636**
21.90	0.4465	0.8086	0.4637	0.5236	0.4568	0.5584	0.5955	0.7593	0.8266	0.5380	0.7414	0.5028	**0.5482**
20.30	0.7635	0.4661	0.3008	0.5635	0.5954	0.6655	0.5174	0.5689	0.4259	0.4993	0.5045	0.8082	**0.54045**

The results are based on the leave one out method. See Table 3 for details.

**Table 6:** Random phase encoding geometry: Mean SSIM results (OFF & ON) over each image pair and median over all pairs

**Sample rate**	**Image1**	**Image2**	**Image3**	**Image4**	**Image5**	**Image6**	**Image7**	**Image8**	**Image9**	**Image 10**	**Image 11**	**Image 12**	**Median**
62.50	0.4977	0.7761	0.8038	0.7710	0.9385	0.3035	0.3178	0.5755	0.8920	0.5611	0.9340	0.7678	**0.7694**
48.50	0.7205	0.4283	0.4978	0.4190	0.5157	0.3792	0.4124	0.4222	0.5150	0.7992	0.6382	0.4757	**0.48675**
40.60	0.8368	0.4455	0.6163	0.3631	0.5303	0.2675	0.6773	0.6491	0.6260	0.6262	0.5328	0.7319	**0.62115**
32.80	0.7470	0.4417	0.5449	0.5325	0.5322	0.2600	0.2553	0.7156	0.4721	0.5817	0.5961	0.3384	**0.53235**
28.10	0.3020	0.5741	0.3409	0.3296	0.3299	0.2593	0.4213	0.7007	0.3138	0.6112	0.4863	0.3430	**0.34195**
26.60	0.2954	0.4523	0.5428	0.4348	0.4034	0.2812	0.2324	0.6164	0.3333	0.4492	0.5069	0.4524	**0.442**
25.00	0.6157	0.4613	0.3285	0.4298	0.3138	0.2766	0.6734	0.4857	0.4460	0.6840	0.3523	0.3663	**0.4379**
21.90	0.5199	0.2968	0.3911	0.3072	0.4502	0.2662	0.2366	0.4205	0.3047	0.3096	0.5264	0.4307	**0.35035**
20.30	0.3137	0.2704	0.4546	0.3005	0.4328	0.3067	0.2069	0.3503	0.2660	0.4082	0.2628	0.3512	**0.3102**

The results are based on the leave one out method. See Table 3 for details.

**Table 7:** Optimal alpha values for dyadic phase encoding geometry (SSIM optimization)

**Sample rate**	**Image1**	**Image2**	**Image3**	**Image4**	**Image5**	**Image6**	**Image7**	**Image8**	**Image9**	**Image 10**	**Image11**	**Image 12**	**Median**
62.50	5.187*1E-04	4.382*1E-04	4.185*1E-04	6.067*1E-04	2.487*1E-04	5.630*1E-04	4.282*1E-04	4.180*1E-04	3.834*1E-04	2.264*1E-04	2.934*1E-04	1.333*1E-04	4.18*1E-04
48.50	4.872*1E-04	7.729*1E-04	3.677*1E-04	4.199*1E-05	7.021*1E-04	3.698*1E-04	2.637*1E-05	3.794*1E-04	1.545*1E-04	5.969*1E-05	6.555*1E-04	3.687*1E-04	3.69*1E-04
40.60	4.577*1E-04	2.174*1E-04	6.738*1E-04	1.573*1E-04	3.090*1E-04	2.712*1E-04	2.207*1E-04	1.597*1E-04	1.580*1E-04	2.485*1E-04	7.190*1E-04	9.229*1E-05	2.35*1E-04
32.80	1.446*1E-04	2.568*1E-04	5.670*1E-04	1.686*1E-04	1.308*1E-04	3.551*1E-04	3.744*1E-04	1.821*1E-04	4.293*1E-04	1.641*1E-04	4.952*1E-04	7.518*1E-04	3.06*1E-04
28.10	4.738*1E-04	2.575*1E-04	1.294*1E-04	1.245*1E-04	9.180*1E-05	4.849*1E-04	1.502*1E-04	3.984*1E-04	4.354*1E-04	5.536*1E-04	6.348*1E-05	4.926*1E-04	3.28*1E-04
26.60	5.961*1E-04	2.208*1E-04	2.764*1E-04	8.724*1E-05	2.422*1E-04	1.694*1E-03	3.760*1E-04	1.450*1E-04	1.747*1E-04	2.461*1E-04	3.492*1E-04	1.405*1E-04	2.44*1E-04
25.00	1.576*1E-04	2.046*1E-04	1.709*1E-05	1.746*1E-04	3.734*1E-04	5.181*1E-04	2.947*1E-04	5.724*1E-04	5.362*1E-04	1.410*1E-04	3.343*1E-04	3.248*1E-04	3.10*1E-04
21.90	2.257*1E-04	6.291*1E-04	1.065*1E-04	1.095*1E-04	2.912*1E-04	5.319*1E-04	2.600*1E-04	1.323*1E-04	4.067*1E-04	6.829*1E-04	9.795*1E-04	3.029*1E-04	2.97*1E-04
20.30	1.563*1E-05	2.289*1E-04	1.435*1E-05	1.641*1E-04	−2.664*1E-05	2.285*1E-04	3.615*1E-04	1.240*1E-04	1.230*1E-04	3.426*1E-05	1.007*1E-05	7.462*1E-06	7.86*1E-05

The optimal values are shown for each image pair. The median value for each sampling geometry is also shown. In the leave one out method, the median values were only computed over the training sets. See Table 3 for details.

**Table 8:** Optimal alpha values for spiral low-pass geometry (SSIM optimization)

**Sample rate**	**Image1**	**Image2**	**Image3**	**Image4**	**Image5**	**Image6**	**Image7**	**Image8**	**Image9**	**Image 10**	**Image11**	**Image 12**	**Median**
62.50	6.742*1E-05	2.440*1E-04	3.171*1E-04	3.987*1E-04	2.606*1E-04	2.529*1E-04	2.591*1E-04	1.550*1E-04	1.728*1E-04	4.957*1E-04	3.440*1E-04	1.499*1E-04	**2.56E-04**
48.50	1.805*1E-04	1.322*1E-04	2.967*1E-04	1.799*1E-04	2.294*1E-04	3.108*1E-04	1.297*1E-04	2.942*1E-04	2.484*1E-04	4.276*1E-04	4.420*1E-04	1.798*1E-04	**2.39E-04**
40.60	2.994*1E-04	1.038*1E-06	2.844*1E-04	1.086*1E-04	2.632*1E-04	2.026*1E-04	3.734*1E-04	2.843*1E-04	6.503*1E-04	7.424*1E-05	1.903*1E-04	2.900*1E-04	**2.74E-04**
32.80	2.261*1E-05	9.766*1E-07	2.617*1E-04	1.784*1E-05	1.651*1E-04	7.080*1E-05	6.673*1E-05	1.561*1E-04	1.329*1E-05	4.016*1E-05	9.338*1E-05	5.145*1E-05	**5.91E-05**
28.10	5.980*1E-06	0.0E + 00	7.971*1E-05	1.282*1E-06	2.110*1E-05	8.163*1E-05	2.121*1E-04	9.640*1E-06	3.081*1E-06	1.708*1E-05	5.890*1E-06	1.438*1E-06	**7.81E-06**
26.60	3.779*1E-06	−2.441*1E-07	8.136*1E-05	9.766*1E-07	2.803*1E-05	1.820*1E-04	3.906*1E-06	7.959*1E-06	9.220*1E-06	1.842*1E-05	6.043*1E-06	4.747*1E-05	**8.59E-06**
25.00	3.829*1E-06	0.0E + 00	1.626*1E-04	3.052*1E-08	9.683*1E-05	1.134*1E-04	6.404*1E-05	4.971*1E-05	5.096*1E-06	1.678*1E-05	5.286*1E-06	4.147*1E-05	**2.91E-05**
21.90	1.008*1E-05	3.052*1E-08	2.252*1E-05	4.578*1E-08	9.543*1E-05	7.703*1E-05	1.178*1E-04	3.884*1E-05	3.132*1E-05	1.179*1E-05	1.053*1E-05	2.166*1E-05	**2.21E-05**
20.30	5.893*1E-06	−5.341*1E-08	1.347*1E-05	2.441*1E-07	9.809*1E-06	3.146*1E-05	3.906*1E-06	4.454*1E-05	6.435*1E-05	3.173*1E-05	1.164*1E-05	4.158*1E-05	**1.26E-05**

The optimal values are shown for each image pair. The median value for each sampling geometry is also shown. In the leave one out method, the median values were only computed over the training sets. See Table 3 for details.

**Table 9:** Optimal alpha values for random sampling on PDF geometry (SSIM optimization)

**Sample rate**	**Image1**	**Image2**	**Image3**	**Image4**	**Image5**	**Image6**	**Image7**	**Image8**	**Image9**	**Image 10**	**Image11**	**Image 12**	**Median**
62.50	2.704*1E-04	3.669*1E-04	3.817*1E-04	3.978*1E-04	2.543*1E-04	4.777*1E-04	3.090*1E-04	2.522*1E-04	4.710*1E-04	5.479*1E-04	2.637*1E-04	2.531*1E-04	3.38*1E-04
48.50	1.672*1E-03	2.404*1E-03	2.460*1E-03	1.266*1E-03	0E + 00	2.185*1E-03	1.924*1E-03	1.627*1E-03	1.606*1E-03	8.298*1E-04	1.865*1E-03	1.848*1E-03	1.76*1E-03
40.60	1.098*1E-03	2.381*1E-03	1.467*1E-03	2.400*1E-03	4.761*1E-06	−5.186*1E-05	1.389*1E-03	1.380*1E-03	1.284*1E-03	2.162*1E-03	1.273*1E-03	3.481*1E-03	1.38*1E-03
32.80	1.201*1E-03	0E + 00	1.190*1E-03	1.107*1E-03	1.484*1E-03	1.959*1E-03	1.928*1E-03	1.174*1E-03	1.759*1E-03	1.375*1E-03	1.003*1E-03	2.092*1E-03	1.29*1E-03
28.10	3.753*1E-03	9.521*1E-06	4.616*1E-03	9.082*1E-04	2.677*1E-03	−6.645*1E-05	1.673*1E-03	1.933*1E-03	2.526*1E-03	1.460*1E-03	2.445*1E-03	3.229*1E-03	2.19*1E-03
26.60	1.980*1E-03	1.522*1E-03	1.535*1E-03	1.040*1E-03	1.393*1E-03	5.993*1E-04	6.140*1E-04	8.419*1E-04	1.679*1E-03	1.061*1E-03	1.171*1E-03	1.469*1E-03	1.28*1E-03
25.00	8.867*1E-04	4.137*1E-04	1.997*1E-03	1.074*1E-05	6.785*1E-04	1.424*1E-03	2.551*1E-03	1.506*1E-03	4.349*1E-04	7.230*1E-04	4.732*1E-04	1.346*1E-03	8.05*1E-04
21.90	1.003*1E-03	1.731*1E-03	1.562*1E-03	2.544*1E-03	1.717*1E-03	1.979*1E-03	1.400*1E-03	2.576*1E-04	2.223*1E-03	9.923*1E-04	9.351*1E-04	2.016*1E-03	1.64*1E-03
20.30	1.388*1E-03	2.289*1E-03	2.344*1E-05	1.739*1E-03	3.741*1E-03	2.142*1E-03	2.476*1E-03	1.484*1E-03	−6.022*1E-05	−2.979*1E-05	1.257*1E-03	1.934*1E-03	1.61*1E-03

The optimal values are shown for each image pair. The median value for each sampling geometry is also shown. In the leave one out method, the median values were only computed over the training sets. See Table 3 for details.

**Table 10:** Optimal alpha values for random phase encoding (SSIM optimization)

**Sample rate**	**Image1**	**Image2**	**Image3**	**Image4**	**Image5**	**Image6**	**Image7**	**Image8**	**Image9**	**Image 10**	**Image11**	**Image 12**	**Median**
62.50	3.432*1E-04	5.724*1E-04	1.449*1E-04	2.051*1E-04	−3.137*1E-05	7.519*1E-04	4.396*1E-04	4.749*1E-04	−5.880*1E-06	2.859*1E-04	7.813*1E-06	6.028*1E-04	3.15*1E-04
48.50	−1.633*1E-06	4.126*1E-04	−2.972*1E-05	−6.086*1E-05	−1.590*1E-05	−1.863*1E-04	−1.957*1E-04	4.404*1E-04	−5.236*1E-05	−2.679*1E-04	7.813*1E-06	5.633*1E-04	−2.28*1E-05
40.60	9.952*1E-04	4.536*1E-04	2.723*1E-04	2.109*1E-04	2.979*1E-05	2.140*1E-04	1.763*1E-04	4.544*1E-04	5.251*1E-04	4.368*1E-04	−6.852*1E-05	8.507*1E-04	3.55*1E-04
32.80	5.493*1E-07	−4.938*1E-04	−6.073*1E-06	−1.334*1E-04	−5.382*1E-05	5.497*1E-04	2.771*1E-05	−7.382*1E-05	−4.323*1E-05	−4.177*1E-04	−3.918*1E-05	−5.866*1E-04	−4.85*1E-05
28.10	−3.632*1E-05	2.559*1E-04	−3.033*1E-05	−1.074*1E-05	−3.271*1E-05	4.256*1E-04	7.668*1E-04	1.416*1E-04	−7.233*1E-06	3.029*1E-06	−1.099*1E-05	6.348*1E-06	−2.10*1E-06
26.60	−3.906*1E-06	1.526*1E-07	−3.009*1E-05	−3.311*1E-05	−4.181*1E-05	2.896*1E-05	2.068*1E-03	−4.675*1E-05	−5.447*1E-06	−3.906*1E-06	5.127*1E-06	−7.563*1E-05	−4.68*1E-06
25.00	−1.844*1E-04	−4.150*1E-06	−1.160*1E-04	−4.858*1E-05	8.374*1E-05	1.191*1E-03	6.500*1E-04	1.036*1E-04	9.277*1E-06	2.930*1E-06	−7.629*1E-08	−1.639*1E-05	1.43*1E-06
21.90	9.766*1E-07	7.047*1E-04	1.367*1E-05	1.121*1E-03	3.906*1E-06	2.021*1E-03	6.571*1E-03	9.662*1E-04	1.068*1E-06	9.479*1E-05	1.271*1E-03	7.481*1E-04	7.26*1E-04
20.30	1.318*1E-04	2.374*1E-05	2.148*1E-05	6.586*1E-05	2.138*1E-04	5.205*1E-06	1.572*1E-06	1.978*1E-05	6.895*1E-06	4.661*1E-04	4.562*1E-05	4.753*1E-04	3.47*1E-05

The optimal values are shown for each image pair. The median value for each sampling geometry is also shown. In the leave one out method, the median values were only computed over the training sets. See Table 3 for details.

**Table 11:** Optimal beta values for dyadic phase encoding geometry (SSIM optimization)

**Sample rate**	**Image1**	**Image2**	**Image3**	**Image4**	**Image5**	**Image6**	**Image7**	**Image8**	**Image9**	**Image 10**	**Image11**	**Image 12**	**Median**
62.50	1.084*1E-03	2.698*1E-04	4.536*1E-04	−7.898*1E-05	2.957*1E-04	3.546*1E-04	1.299*1E-04	5.352*1E-04	3.703*1E-04	6.006*1E-04	5.883*1E-04	2.869*1E-04	3.62*1E-04
48.50	5.988*1E-04	2.676*1E-04	3.268*1E-04	3.989*1E-04	5.205*1E-04	5.235*1E-04	3.940*1E-04	3.740*1E-04	5.166*1E-04	1.891*1E-04	2.092*1E-04	3.794*1E-04	3.87*1E-04
40.60	9.831*1E-04	2.217*1E-04	1.020*1E-03	1.729*1E-04	5.130*1E-04	2.909*1E-04	−5.859*1E-05	4.841*1E-04	5.129*1E-04	4.080*1E-04	7.499*1E-04	5.183*1E-04	4.99*1E-04
32.80	4.906*1E-04	4.087*1E-04	4.010*1E-04	7.449*1E-04	7.143*1E-04	4.673*1E-04	1.331*1E-03	5.408*1E-04	1.058*1E-03	7.070*1E-04	6.733*1E-04	4.987*1E-04	6.07*1E-04
28.10	8.401*1E-04	2.506*1E-04	4.290*1E-04	4.456*1E-04	6.025*1E-04	2.600*1E-04	5.224*1E-04	5.955*1E-04	2.392*1E-04	1.120*1E-03	6.011*1E-04	1.297*1E-03	5.59*1E-04
26.60	2.075*1E-03	3.907*1E-04	3.184*1E-04	2.024*1E-03	5.989*1E-04	1.206*1E-03	7.061*1E-04	2.813*1E-04	4.880*1E-04	6.309*1E-04	6.032*1E-04	7.237*1E-04	6.17*1E-04
25.00	5.646*1E-04	3.037*1E-04	3.977*1E-04	6.276*1E-04	6.339*1E-04	2.351*1E-04	1.793*1E-04	9.519*1E-04	4.954*1E-04	4.382*1E-04	9.376*1E-04	7.264*1E-04	5.30*1E-04
21.90	1.004*1E-03	1.034*1E-03	1.033*1E-03	2.980*1E-04	9.097*1E-04	9.206*1E-04	1.079*1E-03	7.043*1E-04	−9.717*1E-05	1.310*1E-03	1.435*1E-03	7.850*1E-04	9.62*1E-04
20.30	4.453*1E-04	6.589*1E-04	3.739*1E-04	5.820*1E-04	1.353*1E-03	4.033*1E-04	8.078*1E-04	7.510*1E-04	5.616*1E-04	1.066*1E-03	2.823*1E-04	2.136*1E-04	5.72*1E-04

The optimal values are shown for each image pair. The median value for each sampling geometry is also shown. In the leave one out method, the median values were only computed over the training sets. See Table 3 for details.

**Table 12:** Optimal beta values for spiral low-pass geometry (SSIM optimization)

**Sample rate**	**Image1**	**Image2**	**Image3**	**Image4**	**Image5**	**Image6**	**Image7**	**Image8**	**Image9**	**Image 10**	**Image11**	**Image 12**	**Median**
62.50	2.929*1E-04	4.407*1E-04	5.365*1E-04	3.320*1E-04	5.587*1E-04	4.524*1E-04	4.398*1E-04	4.448*1E-04	1.932*1E-04	8.479*1E-04	4.432*1E-04	1.973*1E-04	4.42*1E-04
48.50	4.101*1E-04	3.171*1E-04	2.837*1E-04	3.176*1E-04	5.204*1E-04	4.530*1E-04	4.610*1E-04	5.018*1E-04	4.352*1E-04	9.829*1E-04	6.130*1E-04	2.600*1E-04	4.44*1E-04
40.60	5.247*1E-04	2.430*1E-04	2.495*1E-04	4.220*1E-04	5.691*1E-04	2.830*1E-04	7.014*1E-04	3.369*1E-04	7.813*1E-04	1.039*1E-04	3.052*1E-04	1.489*1E-04	3.21*1E-04
32.80	3.198*1E-05	3.418*1E-06	2.612*1E-04	2.844*1E-04	2.844*1E-04	3.682*1E-04	3.340*1E-04	2.250*1E-04	2.093*1E-05	5.343*1E-05	1.320*1E-04	1.717*1E-04	1.98*1E-04
28.10	9.243*1E-06	7.813*1E-06	4.480*1E-05	7.660*1E-06	3.377*1E-05	3.461*1E-04	3.067*1E-04	1.434*1E-05	1.066*1E-05	1.429*1E-05	7.380*1E-06	5.000*1E-06	1.25*1E-05
26.60	9.680*1E-06	9.888*1E-06	5.209*1E-05	7.324*1E-06	4.948*1E-05	6.414*1E-04	9.766*1E-06	1.607*1E-05	6.538*1E-06	1.148*1E-05	5.924*1E-06	1.136*1E-04	1.07*1E-05
25.00	7.717*1E-06	7.813*1E-06	9.662*1E-05	9.750*1E-06	1.960*1E-04	2.863*1E-04	7.765*1E-05	8.038*1E-05	5.798*1E-06	1.576*1E-05	6.060*1E-06	9.034*1E-05	4.67*1E-05
21.90	1.051*1E-05	9.750*1E-06	1.438*1E-05	5.714*1E-06	2.244*1E-04	1.726*1E-04	1.944*1E-04	7.759*1E-05	7.979*1E-05	1.394*1E-05	1.187*1E-05	4.749*1E-05	3.09*1E-05
20.30	8.590*1E-06	7.473*1E-06	7.542*1E-06	3.784*1E-06	3.100*1E-05	8.866*1E-05	1.953*1E-06	1.166*1E-04	1.164*1E-04	5.748*1E-05	1.123*1E-05	9.008*1E-05	2.11*1E-05

The optimal values are shown for each image pair. The median value for each sampling geometry is also shown. In the leave one out method, the median values were only computed over the training sets. See Table 3 for details.

**Table 13:** Optimal beta values for random sampling on PDF geometry (SSIM optimization)

**Sample rate**	**Image1**	**Image2**	**Image3**	**Image4**	**Image5**	**Image6**	**Image7**	**Image8**	**Image9**	**Image 10**	**Image11**	**Image 12**	**Median**
62.50	3.879*1E-04	4.778*1E-04	3.463*1E-04	2.347*1E-04	2.575*1E-04	5.832*1E-04	3.699*1E-04	2.552*1E-04	6.620*1E-04	6.740*1E-04	2.959*1E-04	3.955*1E-04	3.79*1E-04
48.50	2.957*1E-03	4.742*1E-03	4.121*1E-03	1.513*1E-03	5.078*1E-04	3.129*1E-03	2.873*1E-03	3.145*1E-03	2.784*1E-03	1.926*1E-03	2.829*1E-03	3.197*1E-03	2.92*1E-03
40.60	2.834*1E-03	3.961*1E-03	3.234*1E-03	4.632*1E-03	4.970*1E-04	4.112*1E-04	2.602*1E-03	3.376*1E-03	1.889*1E-03	3.994*1E-03	2.581*1E-03	4.480*1E-03	3.03*1E-03
32.80	2.285*1E-03	2.578*1E-04	2.069*1E-03	2.080*1E-03	2.182*1E-03	2.415*1E-03	2.275*1E-03	2.245*1E-03	3.317*1E-03	3.334*1E-03	1.757*1E-03	2.649*1E-03	2.26*1E-03
28.10	4.355*1E-03	4.886*1E-04	6.020*1E-03	2.377*1E-03	3.631*1E-03	7.985*1E-04	2.793*1E-03	2.806*1E-03	4.510*1E-03	3.320*1E-03	3.175*1E-03	4.770*1E-03	3.25*1E-03
26.60	2.809*1E-03	2.626*1E-03	3.631*1E-03	1.898*1E-03	1.739*1E-03	5.134*1E-04	1.089*1E-03	1.712*1E-03	3.226*1E-03	1.510*1E-03	1.889*1E-03	2.007*1E-03	1.89*1E-03
25.00	2.474*1E-03	9.774*1E-04	4.994*1E-03	6.392*1E-04	1.381*1E-03	3.023*1E-03	4.750*1E-03	2.253*1E-03	7.196*1E-04	2.347*1E-03	1.936*1E-03	3.353*1E-03	2.30*1E-03
21.90	2.254*1E-03	2.776*1E-03	2.575*1E-03	5.158*1E-03	2.319*1E-03	2.012*1E-03	1.015*1E-03	3.837*1E-04	3.279*1E-03	1.706*1E-03	2.336*1E-03	2.642*1E-03	2.33*1E-03
20.30	2.022*1E-03	4.004*1E-03	3.867*1E-04	3.612*1E-03	4.730*1E-03	2.663*1E-03	1.801*1E-03	2.231*1E-03	5.073*1E-04	3.918*1E-04	2.242*1E-03	3.168*1E-03	2.24*1E-03

The optimal values are shown for each image pair. The median value for each sampling geometry is also shown. In the leave one out method, the median values were only computed over the training sets. See Table 3 for details.

**Table 14:** Optimal beta values for random phase encoding (SSIM optimization)

**Sample Rate**	**Image1**	**Image2**	**Image3**	**Image4**	**Image5**	**Image6**	**Image7**	**Image8**	**Image9**	**Image 10**	**Image11**	**Image 12**	**Median**
62.50	7.155*1E-04	7.113*1E-04	5.765*1E-04	8.666*1E-04	5.501*1E-04	−1.006*1E-03	−6.614*1E-04	−2.959*1E-04	3.854*1E-05	5.893*1E-04	0.000	6.427*1E-04	5.63*1E-04
48.50	1.199*1E-05	1.786*1E-03	2.434*1E-04	4.558*1E-04	1.400*1E-04	1.468*1E-03	1.243*1E-03	1.103*1E-03	2.851*1E-04	1.590*1E-03	0.000	1.707*1E-03	7.79*1E-04
40.60	2.279*1E-03	8.726*1E-04	9.424*1E-04	5.586*1E-04	4.216*1E-04	−3.205*1E-04	−4.021*1E-04	1.538*1E-03	1.124*1E-03	7.060*1E-04	1.046*1E-03	1.522*1E-03	9.08*1E-04
32.80	2.561*1E-04	4.782*1E-03	5.028*1E-05	8.742*1E-04	5.847*1E-04	−7.650*1E-04	−1.547*1E-04	4.421*1E-04	2.294*1E-04	3.368*1E-03	2.354*1E-04	4.703*1E-03	3.49*1E-04
28.10	5.722*1E-04	2.451*1E-04	1.896*1E-04	9.717*1E-05	1.935*1E-04	8.850*1E-05	9.451*1E-04	−7.617*1E-05	5.061*1E-05	6.785*1E-05	8.459*1E-05	4.883*1E-07	9.28*1E-05
26.60	9.766*1E-06	7.360*1E-04	1.344*1E-04	2.263*1E-04	3.434*1E-04	5.932*1E-04	1.190*1E-03	5.092*1E-04	2.915*1E-05	9.766*1E-06	−4.639*1E-06	3.732*1E-04	2.85*1E-04
25.00	1.500*1E-03	3.746*1E-04	1.015*1E-03	8.685*1E-04	6.149*1E-04	1.737*1E-03	1.722*1E-03	1.105*1E-03	3.997*1E-04	5.024*1E-04	2.420*1E-04	8.242*1E-04	8.46*1E-04
21.90	7.324*1E-06	1.136*1E-03	−3.906*1E-06	6.226*1E-04	1.953*1E-06	7.024*1E-04	−3.088*1E-03	1.023*1E-03	7.736*1E-06	−1.375*1E-04	2.603*1E-03	1.717*1E-03	3.15*1E-04
20.30	−4.746*1E-05	8.667*1E-06	3.418*1E-05	4.228*1E-05	8.818*1E-05	−9.306*1E-06	−9.048*1E-06	5.127*1E-06	6.141*1E-06	−4.362*1E-04	2.938*1E-05	−3.554*1E-04	5.63*1E-06

The optimal values are shown for each image pair. The median value for each sampling geometry is also shown. In the leave one out method, the median values were only computed over the training sets. See Table 3 for details.

For each sampling geometry, we provide the median *α, β*-values in the last column of Tables 7, 8, 9, 10, 11, 12, 13, 14. The recommendation to use the median *α, β*-values for reconstruction was validated using leave-one-out. Here, note that the mean SSIM values reported in Tables 3, 4, 5, 6 are based on performing the image reconstruction using the median values from the training set of the remaining 11 image pairs. There is thus a mismatch between the median *α, β* values and the optimal values that can only be estimated when the entire image is available. Yet, despite this mismatch, we find that the second approach worked very well. For example, all of the SLP reconstructions for 20.3% sampling gave average SSIM index values above 0.93.

We next discuss the relationship between PSNR and mean SSIM values. Numerically, an SSIM value of 1 corresponds to an infinite value for the PSNR. Furthermore, an SSIM value of 0 corresponds to a negative infinity value for PSNR. Ideally, an increase in the SSIM index will also correspond to an increase in PSNR. We have verified this fact for the mean-SSIM values of Table 4 and their corresponding PSNR values. On the other hand, this property does not hold for individual images. For example, image #2 for sampling at 20.30% has a PSNR value that is above the median, while its SSIM value is below the median. In such cases, the SSIM trend is considered to be superior to the PSNR. Overall though, all of the SLP reconstructions gave mean SSIM values above 0.93. Thus, the SLP geometry reconstructions are considered to be excellent.

## Discussion

We have found that parameter optimization achieved significant image quality improvements using a relatively small number of iterations. Typically, five iterations were required to improve PSNR by over 10 dB from the initial values of *α*=0, *β*=0 and achieve reconstructions that were within 5% of the optimal quality value.

We take a closer look at the *α* and *β* ranges for select geometries from the DPE and SLP classes. We selected both the DPE and SLP geometries with the *least* number of samples that still provided reasonable performance regions in the optimal quality regions for 30 dB, 35 dB, 40 dB, and 45 dB. Examining the range of the parameters provides a more detailed description of the parameter space, and which constraint in (3) is essential for acceptable reconstructions. The minimum and maximum parameter values that define the vertices of the inner and outer bounding boxes are listed in Table 2.

Three instances in the above table stand out because the minimum alpha and/or beta parameters are negative. These values are a by-product of the interpolation and intersection method used to calculate the parameter ranges. When negative parameter values were inserted into the optimization algorithm, the reconstruction algorithm breaks down. In these cases, we replace the negative value with zero to achieve an acceptable result.

It is imperative that our discussion turn to the effect of a zero parameter value for either constraint in the reconstruction problem. Typically, the effect of *α*=0 is a more prominent presence of high-frequency errors in the spatial domain, as well as a higher amount of artifacts from the sampling geometry. Conversely, the effect of *β*=0 is a loss of high-frequency spatial components in the spatial domain reconstruction. In this case, the TV-norm tends to drive the solution to result whose finite difference in each dimension is minimized. While removing one of the penalty terms in the CS objective function may result in a usable reconstruction, our observations indicated that this case will not be optimal.

When both parameters are zero, the proposed optimization framework breaks down. It is possible to achieve acceptable image quality when one of the two parameters is zero, but not both. While completely removing one of the penalty terms in (3) may result in acceptable reconstructions in terms of PSNR, an increased PSNR can be achieved by using the (non-zero) optimal values.

For activity detection, we have found that optimization of the difference image led to excellent results. In particular, the SLP geometry gave excellent average SSIM values of 0.9747 (max = 1). The use of the leave-one-out validation with mean SSIM optimization also verified the fact that the SLP family of geometries gives the best result. Furthermore, recommended parameter values for the SLP geometries are given by the median values of Tables 8 and 12.

## Conclusions

In conclusion, we have found that CS parameter optimization can dramatically improve fMRI image reconstruction quality. Furthermore, deterministic SLP scanning geometries (similar to the 3D geometries of [4]) consistently gave the best image reconstruction results. The implication of this result is that less complex sampling geometries will suffice over random sampling, provided the TV-Norm and Transform penalty parameters are selected from the range of values we have calculated in our proposed methodology. We have also found that we can obtain stable parameter regions that can be used to achieve specific levels of image reconstruction quality when combined with specific k-space sampling geometries. More importantly, median parameter values can be used for obtaining excellent reconstructions. This observation has been validated using leave-one-out and optimization based on the mean SSIM index.

## Competing interests

The authors declare that they have no competing interests.

## Authors' contributions

Dr. J carried out most of the investigation, design, and development. Professor P conceived of the project, participated and advised in the design and coordination of all research activites. Professor C participated in the study design and clinical image collection, and provided clinical guidance and interpreation. All authors read and approved the final manuscript.
